# Ameliorative effect of curcumin and zinc oxide nanoparticles on multiple mechanisms in obese rats with induced type 2 diabetes

**DOI:** 10.1038/s41598-021-00108-w

**Published:** 2021-10-19

**Authors:** Shaymaa Abdulmalek, Asmaa Eldala, Doaa Awad, Mahmoud Balbaa

**Affiliations:** 1 Department of Biochemistry, Faculty of Science, Alexandria University, Alexandria, 21511 Egypt; 2 Center of Excellency for Preclinical Study (CE-PCS), Pharmaceutical and Fermentation Industries Development Centre, The City of Scientific Research and Technological Applications, SRTA-City, New Borg El-Arab City, Alexandria, Egypt

**Keywords:** Biochemistry, Cell biology, Drug discovery, Molecular biology

## Abstract

The present study was carried out to investigate the therapeutic effect of synthesized naturally compounds, curcumin nanoparticles (CurNPs) and metal oxide, zinc oxide nanoparticles (ZnONPs) on a high-fat diet (HFD)/streptozotocin (STZ)-induced hepatic and pancreatic pathophysiology in type 2 diabetes mellitus (T2DM) via measuring AKT pathway and MAPK pathway. T2DM rats were intraperitoneally injected with a low dose of 35 mg/kg STZ after being fed by HFD for 8 weeks. Then the rats have orally received treatments for 6 weeks. HFD/STZ-induced hepatic inflammation, reflected by increased phosphorylation of p38-MAPK pathway’s molecules, was significantly decreased after nanoparticle supplementation. In addition, both nanoparticles significantly alleviated the decreased phosphorylation of AKT pathway. Further, administration of ZnONPs, CurNPs, conventional curcumin, and ZnSO_4_ (zinc sulfate), as well as metformin, effectively counteracted diabetes-induced oxidative stress and inflammation in the internal hepatic and pancreatic tissues. Based on the results of the current study, ZnONPs and CurNPs could be explored as a therapeutic adjuvant against complications associated with T2DM. Both nanoparticles could effectively delay the progression of several complications by activating AKT pathway and down-regulating MAPK pathway. Our findings may provide an experimental basis for the application of nanoparticles in the treatment of T2DM with low toxicity.

## Introduction

Type 2 diabetes mellitus (T2DM) accounts for 90% and 95% of diabetes; with the greatest amounts in low- and middle-income countries and the deaths caused by diabetes and its complications have reached 4.2 million in 2019^1^. Chronic hyperglycemia causes the assembly of oxidative stress, which is related to the increased expression of pro-inflammatory cytokines^2^. In T2DM, the existence of continually elevated glucose levels causes an elevation of the speed of advanced glycation end products (AGEs) that activate a cascade of reactive oxygen species (ROS) production and activation of pro-inflammatory pathways, which act as critical biological factors in the pathogenesis of T2DM^3,4^.

Unfortunately, several oral hypoglycemic drugs displayed anti-diabetic effects through different mechanisms of action with adverse side effects. Therefore, the utilization of medicinal plants has become the main key player of all available therapies due to low cost, ease of availability, and the least side effects. Curcumin is the most effective polyphenol compound in the rhizome of turmeric (*Curcuma longa*). It has strong anti-inflammatory, neuroprotective, hypoglycemic, antioxidant, anticancer, and anti-metastatic efficacies^5,6^. In addition, curcumin exhibits numerous pharmacological activities against many chronic diseases such as T2DM, Alzheimer’s disease and protects against liver injury^7^.

Aside from pure curcumin's limits as a medicinal agent, some studies have discovered dose-dependent toxicity in curcumin linked to DNA damage and chromosomal changes, as well as ulcers, hypoplasia^8^, and iron chelation^9^. Also, some researchers argue that the relatively low incidence of gastrointestinal cancer at higher consumption of turmeric^10^. A considerable number of studies have been published in the last few years to improve curcumin's potency and effectiveness. Curcumin nanoparticles (CurNPs) have been demonstrated to have more biological activity than conventional curcumin and can help in the treatment of a variety of chronic diseases. The major drawback of the effectiveness of curcumin oral administration is the low bioavailability owing to poor gastrointestinal absorption and solubility, rapid metabolism, degradation at intestinal pH, and systemic elimination^11^. Alternative formulations of curcumin as nanoparticles are used to improve the poor biopharmaceutical properties of curcumin and to resist the rapid metabolism and systemic elimination^12^. Recently, several studies reported the use of CurNPs in models of diabetic complications^13,14^, but the effect of CurNPs on several internal signaling pathways is insufficient.

Previously, studies also reported the role of metals in the regulation of glucose metabolism and the association of their deficiency with the risk of diabetes. Particularly, zinc was capable of decreasing the levels of blood glucose, HbA1c, and lipid profiles accompanied by increased AKT activity in diabetic murine models^15^. Zinc is an important co-factor for several antioxidant enzymes found at a high level in the β-cell^16^.

Noticeably, zinc oxide nanoparticles (ZnONPs), as one of the most important metal oxide nanoparticles, have been used as therapeutic agents in many biomedical applications due to their unique physical and chemical properties^17^. Further, the anti-inflammatory and antidiabetic effects of ZnONPs were reported previously as a novel agent to deliver zinc^18,19^. Although ZnONPs have been reported to have adverse effects on the animal; in particular, the impact of this nanoparticle on oxidative stress, inflammation, and internal signaling pathways, studies in this regard are insufficient. As a result, more research is needed to assess their utility as therapeutic agents in chronic therapies. Additionally, the therapeutic efficacy of this nanoparticle in comparison with its bulk counterparts has not been fully investigated. The lack of this knowledge limits the integral control of alterations suffered by the diabetic patient.

Although the antidiabetic activities of CurNPs and ZnONPs were observed in previous studies, the underlying molecular mechanisms, safety doses, and therapeutic efficacies of these nanoparticles were not fully determined. In our previous work, we proved the antidiabetic effect of dietary zinc and curcumin to control diabetes in rats with some toxicity^20^. Upon these bases, our work was designed to evaluate a powerful antidiabetic agent with minimal toxicity using nanotechnology. This can be fulfilled via studying the biochemical and molecular effects of metal oxide nanoparticles, ZnONPs besides natural medicinal nanoparticles, CurNPs in common chronic diseases like T2DM in rats using two doses of ZnONPs and CurNPs, as well as a comparison between these nanoparticles and their conventional forms, zinc sulfate (ZnSO_4_) and curcumin and also an antidiabetic drug, metformin (MT), to find out the most efficient and safe compound(s) and also the effective dose in the treatment of common pathogenic responses developed in diabetes such as oxidative stress, insulin resistance, and inflammation in hepatic and pancreatic tissues. The impact of nano-curcumin and nano-zinc oxide on survival, AKT, and MAPK signaling pathways in hepatic tissue of obese type 2 diabetes-induced rats were also analyzed.

## Material and methods

### Preparation of curcumin and zinc oxide nanoparticles

Curcumin nanoparticles were prepared by dissolving curcumin powder (Sigma Aldrich, USA) in Dichloromethane (Sigma Aldrich, USA) to prepare (5 mg/ml) curcumin stock solution, then 1 ml of the solution was added in dropwise method to (50 ml) boiling water in ultra-sonication condition for about 30 min. The mixture was then stirred at 800 rpm for 20 min until an orange-colored precipitate was obtained. Finally, the obtained pellet was used in the study. While preparation of ZnONPs occurred by dropwise addition of 2 M sodium hydroxide (Sigma Aldrich, USA) to the aqueous solution of 1 M ZnSO_4_ heptahydrate (Sigma Aldrich, USA) after addition of 2 ml 0.01% polyvinyl alcohol then the resulting solution was vigorously stirred for almost 18 h. The white precipitate was filtered and washed with distilled water then dried using a muffle furnace (Gilson, USA) at a temperature of 100 °C for 2 h, then crushed to fine powders and finally calcined at 450 °C.

### Characterization of curcumin and zinc oxide nanoparticles

Curcumin and ZnO nanoparticles were fixed for TEM analysis by dropping a drop of the suspension onto carbon-coated copper grids. After that, the samples were dried with an infrared lamp, and finally, the images were captured with a TEM (JEOL JEM-1400Flash instrument, Japan). Moreover, the average particle size and size distribution, polydispersity index (PDI) of particles were evaluated by Zetasizer (Malvern Instruments, UK), based on the dynamic light scattering (DLS) technique. Additionally, Fourier transforms infrared (FT-IR) spectroscopy (FTIR- 8400 S Shimadzu, Japan) was also performed on both nanoparticles.

### Evaluation of cytotoxic effects of prepared nanoparticles on rat hepatocytes

Hepatocyte isolation from male Sprague–Dawley rats was made consistent with the collagenase perfusion method, cells (1 × 10^6^ cells/ml) were placed into Krebs–Henseleit buffer, pH, 7.4 including HEPES (12.5 mM) (Sigma Aldrich, USA) and held at 37 °C with 95% O_2_ and 5% CO_2_. Hepatocytes with a viability of more than 90% were used in the experiments^21^. For cytotoxicity assay, CurNPs and ZnONPs were introduced into 96-well plates in triplicate at concentrations of 1000, 500, 250, 125, 62.5, 31.25, 15.6, and 7.8 µg/ml. The MTT test was used to assess the number of viable cells after 48 h. Then, the optical density was measured with the microplate reader (SunRise, TECAN, Inc., USA). The percentage of viability was calculated as [(ODt/ODc)] × 100%, where ODc is the mean optical density of untreated cells and ODt is the mean optical density of wells treated with the tested compounds.

### Animals

Seventy-two male Albino rats aging 8 weeks, with average body weight 130 ± 10 g, were obtained from the animal house of the institute of graduate studies and research, Alexandria University, Egypt. The animals were housed in polycarbonate cages (9 groups), each group contains 8 rats (4 rats/cage) in a room with a 12 h day-night cycle, with a temperature of 22 ± 2.0 and humidity of 45% to 46%. The rats were fed with a stable normal pellet diet containing 3% fat, 26% protein, 54% carbohydrate, and 17% vitamins and minerals percentage per 100 gm. During the experimental period, all animals survived in the control group, seven rats have died after STZ injection, and the rest of the rats were sacrificed at the end of the experimental period. This study was carried out following ARRIVE guidelines and all animal experiments were approved by the Ethics Committee of Faculty of Science, Alexandria University, Egypt (AU Approval No. 04181103). All the experimental procedures were performed in accordance with all relevant guidelines and regulations.

### Type 2 diabetes mellitus induction

T2DM was induced by daily supplementation of HFD containing protein 25%, carbohydrate 17%, and fat 58%, as a percentage of total Kcal for 8 weeks. After 8 weeks from HFD, overnight fasted obese rats were intraperitoneally (i.p.) administered a small dose of streptozotocin (STZ) (Calbiochem, Sigma Aldrich, USA) (35 mg/kg body weight, in 0.1 M citrate buffer, pH 4.5). After 72 h of STZ injection, diabetes was reported. The animals with blood glucose concentrations of more than 300 mg/dl will be used for the experiment. The ACCU-CHEK active blood glucose meter was used to measure the blood glucose levels.

### Experimental design and animal treatment

**Figure 1:**
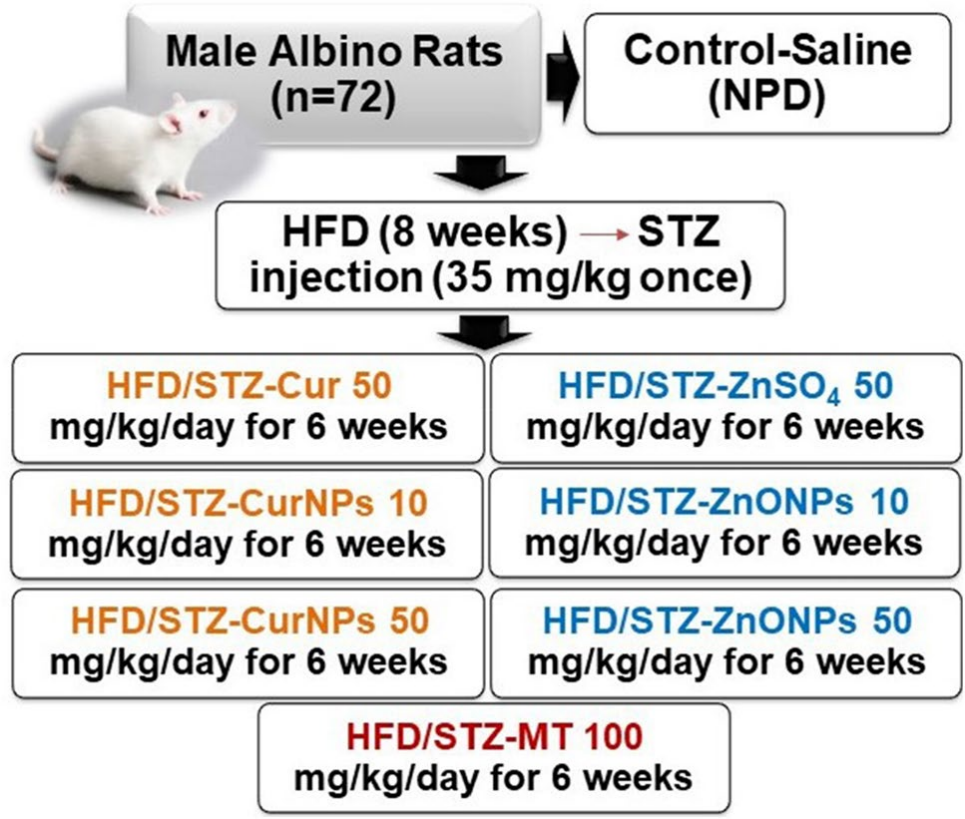
Experimental design and group classification.

Rats were randomly divided into nine groups as shown in Fig. 1. Rats were grouped as follow: control group, where rats were only treated with saline; HFD/STZ-induced group; HFD/STZ-curcumin-treated group (HFD/STZ-cur, 50 mg/kg body weight); HFD/STZ-CurNPs-treated group, 10 and 50 mg/kg body weight; HFD/STZ-ZnSO_4_-treated group, 50 mg/kg body weight; HFD/STZ-ZnONPs-treated group, 10 and 50 mg/kg body weight treated group and HFD/STZ-Metformin-treated group (HFD/STZ-MT, 100 mg/kg body weight). Each group was orally received their respective doses for six weeks. The doses were supplemented at the same time every day and prepared by dissolving the appropriate doses of each therapy in normal saline solution according to the body weight of each rat and then ingested by oral gavage to the rat. At the end of the study, rats were fasted overnight, weighed, and anesthetized using sodium pentobarbital (100 mg/kg, i.p.). The liver and pancreas tissues were quickly removed, washed with ice-cold saline, frozen in liquid nitrogen, and stored at -80 °C. The serum of rats was isolated and stored at − 20 °C.

### Estimation of body weight changes

Throughout the study, the body weight of each rat in each group was reported weekly to estimate body weight changes.

### Liver, Kidney, and lipid profile assays

The levels of serum total cholesterol (TC), triacylglycerol (TG), alanine transaminase (ALT), aspartate transaminase (AST), Gamma-glutamyl transferase (GGT), Urea, and creatinine were examined using commercially available specific kits (Spectrum Diagnostics, Egypt) in accordance with the manufacturer's protocols.

### Assessment of insulin and HOMA-IR

Quantitative measurement of insulin was performed according to ELISA technique using kits specific for rats purchased from (MyBioSource, USA) according to the manufacturer's instructions. Insulin resistance estimation was performed using the homeostasis model assessment method; HOMA-IR, and was calculated by the following formula:

Plasma glucose (mg/dl) × fasting plasma insulin (IU mg/l in the fasting state divided by 405).

the β-cell function was calculated by the following formula:

(HOMA-β) % = [360 × fasting insulin (μIU/ml)]/(fasting glucose (mg/dl) – 63)^22^.

### Measurement of serum Adipokines, AGEs besides hepatic and pancreatic iNOS level

Adiponectin, leptin, and AGEs levels were determined in serum using rat-specific ELISA kits (MyBioSource, USA). In addition, levels of the inflammatory cytokine iNOS were measured in hepatic and pancreatic lysate using a commercially available ELISA kit (MyBioSource, USA) in compliance with the manufacturer's protocols.

### Biomarkers for oxidative stress, enzymatic and non-enzymatic antioxidants

Frozen tissues were homogenized in lysis buffer containing a protease inhibitor (150 mM NaCl, 1% Triton X-100, 10 mM Tris, pH 7.4), the homogenates were centrifuged at 10,000 g for 10 min at 4 °C and the supernatant was separated then total protein contents were measured with the Lowry method^23^. Glutathione peroxidase (GPx), Catalase (CAT), total-Superoxide dismutase (t-SOD), and Glutathione-S-transferase (GST) activities in the liver and pancreas were estimated following previously described method^24–27^. Meanwhile, the concentration of Glutathione reduced (GSH) and total antioxidant capacity (TAC) were determined according to the previously described methods^28,29^. Oxidative stress markers of the liver and pancreas were used to determine lipid peroxidation by measuring thiobarbituric acid reactive substances (TBARS) based on the previously described method^30^. As well, xanthine oxidase (XO) and nitric oxide (NO) were assayed^31,32^.

### Pro-inflammatory cytokines gene expression by quantitative real-time-polymerase chain reaction (qRT-PCR)

Total RNA was isolated from the pancreatic and liver tissues using RNeasy Mini Kit (Qiagen). 1 µg of total RNA was used to produce cDNA using the commercial first-strand cDNA synthesis kit (Thermo Scientific, USA). The cDNA samples were run in triplicate for real-time PCR analysis, and the relative expression of genes related to the internal regulation, β-actin, was measured. The thermal cycling program was as follows; the initial denaturation at 95 °C for 10 min, followed by 40 cycles of 10 s at 95 °C, 10 s at 60 °C, and 20 s at 72 °C. The RNA concentration was determined from the threshold cycle (Ct) values. The mRNA expression levels were calculated relative to the β-actin gene’s mRNA levels using 2^−ΔΔCT^ method^33^. Primer sequence (Sigma Aldrich): **TNF-α** sense: 5′- GCC AGC CTC CGA AGC CAG C-3′, antisense 5′-GGG CGG TAG CGT CCT TGG G-3′. **IL-1β** sense: 5′-TGATGTTCCCATTAGACAGC-3′, antisense 5′-GAGGTGCTGATGTACCAGTT-3′, **β-actin** sense: 5′-TGAGAGGGAAATCGTGCGT-3′, anti-sense 5′-TCATGGATGCCACAGGATTCC-3′.

### Western blotting analyses

Frozen liver tissues 10% (w/v) of the liver was homogenized in lysis buffer containing protease inhibitors (100 mM NaCl, 100 mM EDTA, 0.5% Nonidet p-40, 0.5% Na-deoxycholate, 10 mM Tris, pH 7.5), the homogenate was centrifuged at 2000 g for 10 min at 4 °C and the supernatant was collected for measurement of protein concentration and Western blot. Briefly, 70 µg protein lysates from each sample were mixed with 2X loading buffer (130 mM Tris–HCl, pH 8.0, 30% (v/v) Glycerol, 4.6% (w/v) SDS, 0.02% Bromophenol blue, 2% DTT), boiled for 5 min, and then cooled at 4 °C. Samples were separated on 12% SDS-PAGE and proteins were transferred to a nitrocellulose membrane at 22 V overnight at 4 °C. The membrane was washed three times then incubated in blocking buffer for 1 h at RT and incubated overnight in primary antibodies^34^. The primary antibodies of [β-actin (NB600-501), PI3K p85 alpha (# MA1-74,183), p-PI3K p85 alpha (Tyr508) (# PA5-105,116), p-p38 MAPK (#9215), p38 MAPK (#9211), p-Erk1/2 (#4377), ERK1/2 (#9102), MEK1/2 (#9122), p-MEK1/2 (Ser 217/221; #9154), AKT (#9272), p-AKT (Ser 473; #9271) and, IRS1 [p Tyr612] (NBP1-73,967)] IRS1 (NB100-82,001)] immune-blots were used in this study. After 3 times wash with TBST, the membrane was incubated at RT for 1 h with a secondary antibody. Membranes were washed three times with TBST, and TMB-Blotting solution was used to detect immunoreactive bands, which were then quantified using UVIBAND with image quantification software (version 8, private access).

### Histological analysis

The fixed liver tissues in 10% formalin were processed for dehydration in ascending grades of alcohol, then impregnation. The specimens were then embedded in paraffin and allowed to solidify at room temperature. Using a rotatory microtome, serial sections of 5 μm thick were cut. After that, sections were stained with Haematoxylin and Eosin (H & E) and observed for histopathological changes^35^.

### Statistical analysis

Results are shown as mean ± SEM (standard error of the mean) for eight rats in each group. Significant differences between groups were compared by one-way analysis of variance (ANOVA), followed by Tukey’s posthoc test for multiple comparisons. When p < 0.01, the difference between the groups was considered statistically significant.

## Results

### Characterization of ZnONPs and CurNPs

**Figure 2:**
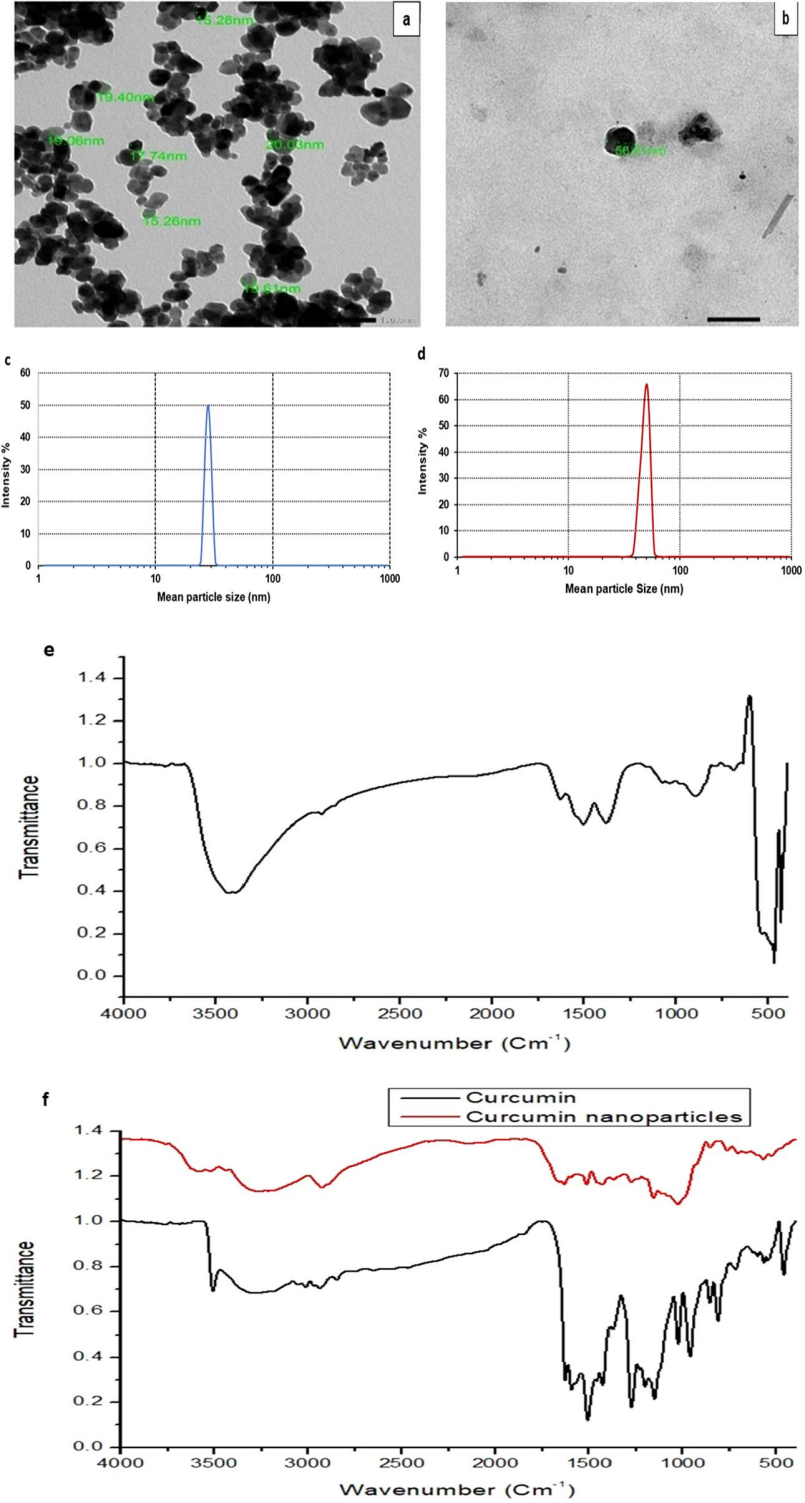
Characterization of prepared ZnONPs and CurNPs. (**a**) TEM analysis of ZnONPs; (**b**) TEM analysis of CurNPs; (**c**) Particle size of ZnONPs; (**d**) Particle size of CurNPs; (**e**) FTIR of ZnONPs and (**f**) FTIR of CurNPs.

ZnONPs had a spherical appearance and most of the nanoparticles had a diameter between 15 and 20 nm (Fig. 2a). Moreover, CurNPs were also spherical, with a diameter of 56 nm (Fig. 2b). In addition, the particle size of ZnONPs (Fig. 2c) and CurNPs (Fig. 2d) were 28.2 and 50.7 nm, respectively confirming the results obtained from TEM analysis. Where the PDI was (0.116 ± 0.01) for ZnONPs and (0.254 ± 0.03) for CurNPs. Further, FTIR spectra of ZnONPs had chain absorption peaks between 400 and 4000 cm^−1^. The absorption peaks located at around 1631, 1505, and 1381 cm^−1^ represent the C=C stretch of alkenes or the C=O stretch of amides. The bands at 1076 are attributed to stretching vibrations of the C–O bond. Peaks at 692 and 899 cm^−1^ may also be related to C-N stretching amine groups. Furthermore, at the absorption peaks of 3433 and 3396 cm^−1^, hydroxyl group stretching can be seen. The major absorption Bands of 422, 434, 470 were allotted and characteristic to the Zn–O bond (Fig. 2e). Curcumin and nano curcumin FTIR spectra were scanned in the mid-infrared region (4000–400 cm^−1^). The stretching vibration of hydrogen-bonded OH found in curcumin correlates to the intense band at wavenumber 3508 cm^−1^. Asymmetric stretching vibrations of C_sp_^2^ − H and C_sp_^3^ − H bonds produce bands at 2939 and 2847 cm^−1^, respectively. The aromatic CH frequency is found to be 1628 cm^−1^. The band at 1427 cm^−1^ corresponds to the aromatic stretching vibrations of the benzene ring. The stretching vibration of the conjugated carbonyl (C=O) is represented by the powerful characteristic band centered at 1507 cm^−1^. The bands centered at 1594 and 1628 cm^−1^ are caused by double-bonded carbon stretching vibrations, whereas the bands centered at 1277 cm^−1^ and 1153 cm^−1^ are caused by C_sp_^2^ − O and C_sp_^3^ − O bonds, respectively. Considering curcumin nanoparticles, the stretching vibration of hydrogen-bonded OH found in nano curcumin correlates to the broad, strong band at wavenumbers 3268 and 3268 cm^−1^. At 2928 cm^−1^, asymmetric stretching vibrations of C_sp_^2^ − H are shown. C−H frequency of the aromatic overtone is obtained at 1633 cm^−1.^ The aromatic stretching vibrations of the benzene ring are shown by bands at 1455 and 1430 cm^−1^. The stretching vibration of the conjugated carbonyl (C=O) is represented by the powerful characteristic band centered at 1513 cm^−1^. Stretching vibrations of C=C, C_sp_^2^ − O, and C_sp_^3^ − O bonds are shown at 1598, 1276, and 1157 cm^−1^, respectively (Fig. 2f).

### Evaluation of cytotoxicity of CurNPs and ZnONPs against rat hepatocytes cell line by MTT assay

The toxicity of biosynthesized CurNPs and ZnONPs was examined in rat hepatocytes cell line. The tested CurNPs exhibited effective activity in the hepatocytes. Increasing concentrations of CurNPs had no significant harmful effect on normal hepatocyte cells. Additionally, the cell viability started to decrease at a high concentration of CurNPs; 1000 µg/ml to be 81%. These data reveal that the CurNPs are non-toxic for this type of healthy cell (Table 1). As well, with increasing the concentration of ZnONPs, there is no significant harmful effect on hepatocyte cells. But the cell viability started to decrease at high concentrations, 500 and 1000 µg/ml to be 79% and 73%, respectively. As a result, ZnONPs are non-toxic to healthy hepatocyte cells but may be toxic at high concentrations (Table 1).

**Table 1:** Evaluation of cytotoxicity of CurNPs and ZnONPs against rat hepatocytes cell line by MTT assay.

Sample concentration (µg/ml)	Mean Viability%	
	CurNPs	ZnONPs
0	100	100
7.8	100	100
15.6	100	100
31.25	100	98.73 ± 0.39
62.5	99.85 ± 0.19	94.66 ± 0.82
125	95.76 ± 1.23	91.40 ± 1.36
250	93.41 ± 0.63	86.13 ± 2.39
500	87.78 ± 1.64	79.98 ± 2.86
1000	81.42 ± 3.76	73.98 ± 2.34

Values are expressed as mean ± SEM.

### Effect of CurNPs and ZnONPs administration on Serum Glucose, Insulin, HOMA-IR, HOMA-β, and AGEs levels of HFD/STZ-induced diabetes

Finding an applicable treatment for hyperglycemia is a topmost priority in diabetes studies. We, therefore, examined whether CurNPs and ZnONPs might have an anti-hyperglycemic effect in diabetic rats. As shown in Table 2, in comparison to control animals, the levels of fasting blood glucose, serum AGEs, serum insulin, and HOMA-IR were found to be increased after induction with HFD/STZ and it remained high during the entire period of the study (*p* < 0.01). By contrast, the level of fasting blood glucose in the healthy rats was within the normal range. The high levels of fasting glucose, AGEs, insulin, and HOMA-IR in induced rats were reduced due to the administration of two doses of CurNPs and ZnONPs as well as conventional curcumin, ZnSO_4_, and metformin for 6 weeks compared to the untreated group (*p* < 0.01). Conversely, beta-cell function showed a marked reduction in HFD/STZ-induced rats relative to the control group (*p* < 0.01), while all current diabetes treatment options exhibited a marked elevation in beta cell function compared to untreated rats (*p* < 0.01).

**Table 2:** Effect of CurNPs and ZnONPs administration on fasting blood glucose, serum fasting insulin, HOMA-IR, HOMA-β, and AGEs levels of HFD/STZ-induced diabetic and control rats.

Groups	Fasting blood glucose (mg/dl)	Serum fasting insulin (µIU/ml)	HOMA-IR	HOMA-β	Serum AGEs (pg/ml)
Control	98.60 ± 1.03^c^	24.30 ± 0.10^e^	5.91 ± 0.06^d^	246.64 ± 8.0^ h^	11.33 ± 0.50^d^
HFD/STZ	481.00 ± 0.71^a^	180.44 ± 0.43^a^	214.30 ± 0.68^a^	155.40 ± 0.39^i^	26.21 ± 0.78^a^
HFD/STZ-Cur	109.20 ± 0.86^b^	80.44 ± 0.15^b^	21.68 ± 0.17^b^	627.66 ± 11.68^ g^	17.24 ± 0.51^b,c^
HFD/STZ-CurNPs-10	75.00 ± 0.71^e^	70.32 ± 0.20^c^	13.02 ± 0.10^c^	2140.85 ± 133.58^b^	13.26 ± 0.65^d^
HFD/STZ-CurNPs-50	79.80 ± 0.58^d,e^	78.60 ± 0.31^b^	15.48 ± 0.14^c^	1692.45 ± 58.52^d^	16.98 ± 0.37^c^
HFD/STZ- ZnSO_4_	76.60 ± 0.93^e^	66.99 ± 0.07^d^	12.67 ± 0.15^c^	1808.50 ± 128.84^c^	19.43 ± 0.45^b^
HFD/STZ-ZnONPs-10	98.00 ± 0.71^c^	81.70 ± 0.20^b^	19.76 ± 0.16^b^	841.70 ± 17.00f.	18.33 ± 0.37^b^
HFD/STZ-ZnONPs-50	83.00 ± 0.71^d^	77.96 ± 0.14^b^	15.97 ± 0.12^c^	1410.68 ± 51.42^e^	15.01 ± 0.74^c^
HFD/STZ-MT	74.00 ± 0.71^e^	71.26 ± 0.09^c^	13.02 ± 0.13^c^	2371.73 ± 154.84^a^	20.33 ± 0.42^b^

Values are expressed as mean ± SEM (n = 7), means for the same parameter with different letters in each column are significantly different (Tukey, *p* < 0.01).

### Effect of CurNPs and ZnONPs administration on serum lipid profile of HFD/STZ-induced rats

Hyperlipidemia was shown after HFD/STZ induction by a marked reduction in HDL-c concentration with a significant elevation in serum TC, TG, LDL-c in comparison with control rats (*p* < 0.01). Administration of two doses of CurNPs and ZnONPs as well as conventional curcumin, ZnSO_4,_ and metformin to HFD/STZ-induced rats produced a marked decline in TC, TG, LDL-c levels along with a marked increase in HDL-c level compared to the untreated group (*p* < 0.01). Besides, better results were obtained for the groups treated with 10 mg/kg CurNPs and 50 mg/kg ZnONPs, which showed a more significant decrease in serum TC, TG, and LDL-c levels than other doses of CurNPs and ZnONPs (Table 3). Furthermore, the groups treated with 10 mg/kg CurNPs and 50 mg/kg ZnONPs exhibited a more significant reduction in serum TC, TG, and LDL-c levels approaching control values than the groups treated with conventional curcumin or 50 mg/kg CurNPs and conventional ZnSO4 or 10 mg/kg ZnONPs.

**Table 3:** Effect of CurNPs and ZnONPs administration on serum lipid profile TC, TG, HDL-c and LDL levels of STZ-induced diabetic and control rats.

Groups	TC (mg/dl)	HDL-c (mg/dl)	TG (mg/dl)	LDL-c (mg/dl)
Control	80.6 ± 0.68^e^	59.80 ± 0.58^a^	92.60 ± 0.93^ h^	2.28 ± 0.67f.
HFD/STZ	532.80 ± 1.66^a^	24.80 ± 0.66^d^	391.00 ± 0.71^a^	229.80 ± 1.16^a^
HFD/STZ-Cur	161.00 ± 0.71^b^	41.00 ± 0.71^b,c^	201.00 ± 0.71^c^	79.80 ± 0.91^b^
HFD/STZ-CurNPs-10	70.00 ± 0.71f.	40.00 ± 0.71^c^	122.00 ± 0.71f.	5.60 ± 0.49^e^
HFD/STZ-CurNPs-50	74.80 ± 0.86f.	41.40 ± 0.93^b,c^	131.00 ± 0.71^e^	7.20 ± 0.42^d^
HFD/STZ- ZnSO_4_	109.20 ± 2.35^c^	43.60 ± 1.03^b^	217.80 ± 0.86^b^	22.04 ± 2.54^c^
HFD/STZ-ZnONPs-10	90.60 ± 0.93^d^	42.00 ± 0.71^b^	110.00 ± 0.71^ g^	26.60 ± 0.76^c^
HFD/STZ-ZnONPs-50	70.00 ± 0.71f.	42.60 ± 0.93^b^	119.80 ± 0.86^ g^	3.44 ± 1.09f.
HFD/STZ-MT	83.00 ± 0.71^e^	45.00 ± 0.71^b^	144.00 ± 0.71^d^	9.20 ± 0.97^d^

Values are expressed as mean ± SEM (n = 7), means for the same parameter with different letters in each column are significantly different (Tukey, *p* < 0.01).

### Effect of CurNPs and ZnONPs administration on serum liver profile of HFD/STZ-induced rats

As shown in Table 4, a significant elevation in serum ALT, AST, and GGT activities in HFD/STZ-induced rats compared to healthy rats (*p* < 0.01). A reverse pattern was exhibited when HFD/STZ-induced group was treated by CurNPs and ZnONPs as well as conventional curcumin, ZnSO_4,_ and metformin. The treated groups exhibited a significant reduction in serum ALT, AST and GGT compared to the untreated group (*p* < 0.01). In comparison to control rats, serum albumin and total protein were found to be non-significantly decreased in HFD/STZ-induced rats (*p* < 0.01). Administration of two doses of CurNPs and ZnONPs as well as conventional curcumin, ZnSO_4,_ and metformin to HFD/STZ-induced rats produced a non-significant change in total protein and albumin concentrations compared to the untreated group, all values within the normal range (*p* < 0.01, Table 5). Administration of curcumin, 10 mg/kg CurNPs and two doses of ZnONPs, as well as metformin to HFD/STZ-induced rats, produced a significant increase in total protein concentration compared to the untreated group with values more than the control group (*p* < 0.01, Table 5).

**Table 4:** Effect of CurNPs and ZnONPs administration on serum liver function enzymes (ALT, AST, and GGT levels) of STZ-induced diabetic and control rats.

Groups	ALT (U/L)	AST (U/L)	GGT (U/L)
Control	32.60 ± 0.93^d^	32.00 ± 0.71f.	11.00 ± 0.71^d^
HFD/STZ	212.60 ± 0.93^a^	243.61 ± 1.21^a^	52.20 ± 0.86^a^
HFD/STZ-Cur	61.40 ± 0.93^b^	53.60 ± 1.03^d^	20.80 ± 0.58^b,c^
HFD/STZ-CurNPs-10	31.80 ± 0.58^d^	42.40 ± 0.93^e^	20.60 ± 0.51^b,c^
HFD/STZ-CurNPs-50	32.40 ± 0.93^d^	40.00 ± 0.71^e^	19.80 ± 0.37^c^
HFD/STZ- ZnSO_4_	57.00 ± 1.41^b^	72.60 ± 0.93^c^	23.00 ± 1.00^b^
HFD/STZ-ZnONPs-10	32.00 ± 0.71^d^	36.00 ± 0.71f.	23.00 ± 0.71^b^
HFD/STZ-ZnONPs-50	36.40 ± 1.36^c^	37.00 ± 0.71f.	21.00 ± 0.71^b^
HFD/STZ-MT	31.00 ± 0.71^d^	87.80 ± 0.86^b^	19.60 ± 0.51^c^

Values are expressed as mean ± SEM (n = 7), means for the same parameter with different letters in each column are significantly different (Tukey, *p* < 0.01).

**Table 5:** Effect of CurNPs and ZnONPs administration on serum liver proteins (albumin and total protein) of STZ-induced diabetic and control rats.

Groups	Albumin (g/dl)	Total protein (g/ml)
Control	3.56 ± 0.13	7.09 ± 0.03^a,b^
HFD/STZ	3.30 ± 0.07	6.10 ± 0.07^b^
HFD/STZ-Cur	2.46 ± 0.09	8.05 ± 0.02^a^
HFD/STZ-CurNPs-10	3.20 ± 0.07	8.20 ± 0.07^a^
HFD/STZ-CurNPs-50	3.36 ± 0.14	7.18 ± 0.07^a^
HFD/STZ- ZnSO_4_	3.20 ± 0.07	7.26 ± 0.09^a^
HFD/STZ-ZnONPs-10	2.96 ± 0.05	8.25 ± 0.10^a^
HFD/STZ-ZnONPs-50	3.40 ± 0.11	8.52 ± 0.12^a^
HFD/STZ-MT	2.90 ± 0.07^a^	8.05 ± 0.05^a^

Values are expressed as mean ± SEM (n = 7), means for the same parameter with different letters in each column are significantly different (Tukey, *p* < 0.01).

### CurNPs and ZnONPs administration improves serum kidney profile

The current results showed a significant increase in serum creatinine and urea levels in HFD/STZ-induced rats compared to control animals (*p* < 0.01). Administration of two doses of CurNPs and ZnONPs as well as conventional curcumin, ZnSO_4,_ and metformin to HFD/STZ-induced rats produced a significant reduction in creatinine and urea levels, within the normal range values compared to the untreated group (*p* < 0.01, Table 6).

**Table 6:** Effect of CurNPs and ZnONPs administration on serum kidney profile (creatinine and urea) of STZ-induced diabetic and control rats.

Groups	Creatinine (mg/dl)	Urea (mg/dl)
Control	0.40 ± 0.01^c^	13.80 ± 0.86^d^
HFD/STZ	1.80 ± 0.03^a^	96.40 ± 1.50^a^
HFD/STZ-Cur	0.71 ± 0.01^b^	29.00 ± 0.71^b^
HFD/STZ-CurNPs-10	0.93 ± 0.01^b^	17.00 ± 0.71^c^
HFD/STZ-CurNPs-50	0.74 ± 0.01^b^	15.80 ± 1.16^d^
HFD/STZ- ZnSO_4_	0.81 ± 0.01^b^	31.00 ± 0.71^b^
HFD/STZ-ZnONPs-10	0.80 ± 0.01^b^	14.20 ± 1.07^d^
HFD/STZ-ZnONPs-50	0.70 ± 0.01^b^	15.80 ± 1.39^d^
HFD/STZ-MT	0.71 ± 0.01^b^	20.60 ± 0.51^c^

Values are expressed as mean ± SEM (n = 7), means for the same parameter with different letters in each column are significantly different (Tukey, *p* < 0.01).

### Effect of CurNPs and ZnONPs on body weight in experimental rats

**Figure 3:**
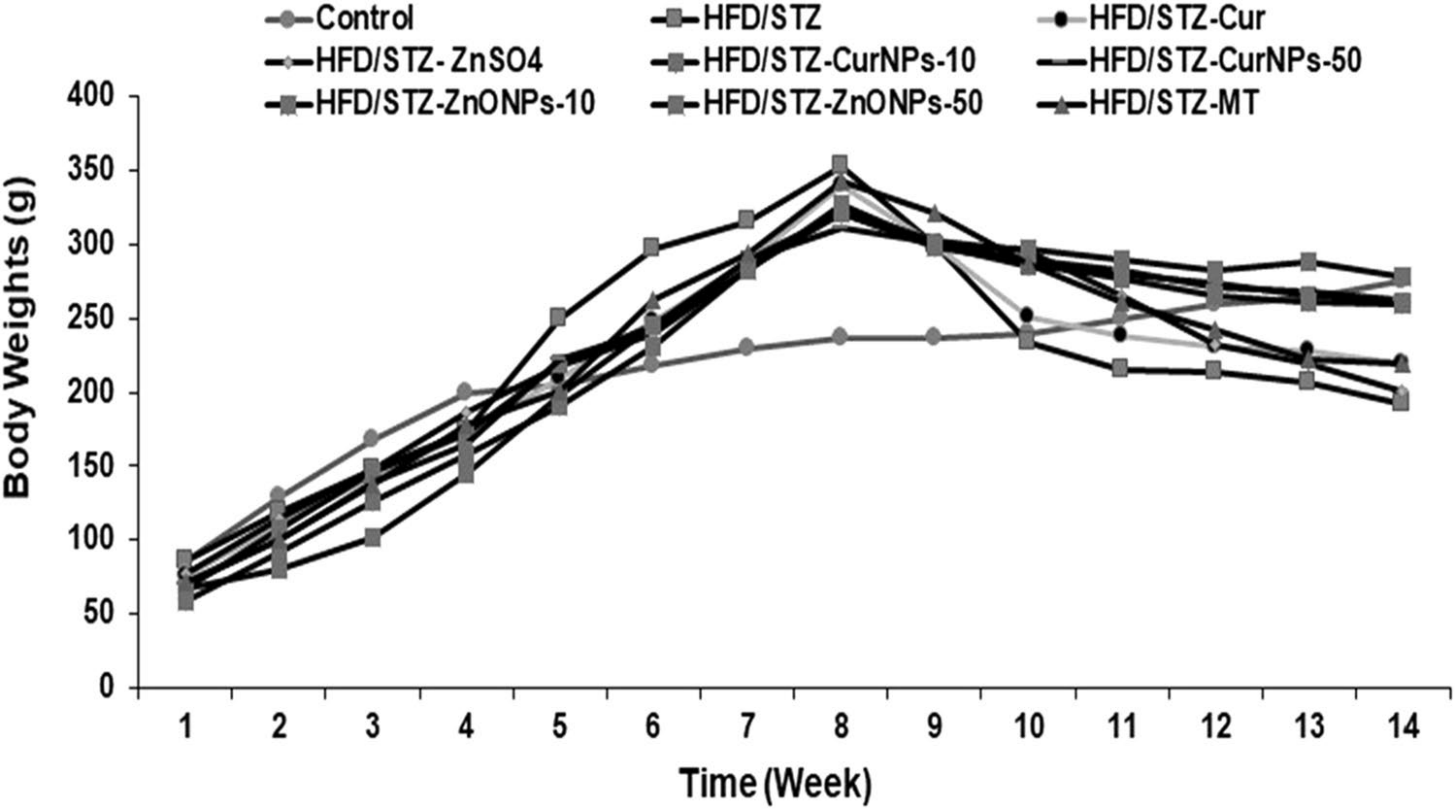
Bodyweight changes for 14 weeks. Body weights of the control group, HFD/STZ-induced rats, and post-administration of CurNPs and ZnONPs, expressed as grams.

At the beginning of the experiment, the mean body weight values across the groups were similar. There was a continuous normal body weight gain in the control groups. Higher body weight was shown in the HFD group after 8 weeks of supplementation, and a loss of body weight was observed after STZ-induction throughout the experiment. Nevertheless, ZnONPs and CurNPs administration prevented body weight loss throughout the experiment compared with untreated groups (Fig. 3).

### CurNPs and ZnONPs recover oxidant and antioxidant balance in hepatic and pancreatic tissues

**Figure 4:**
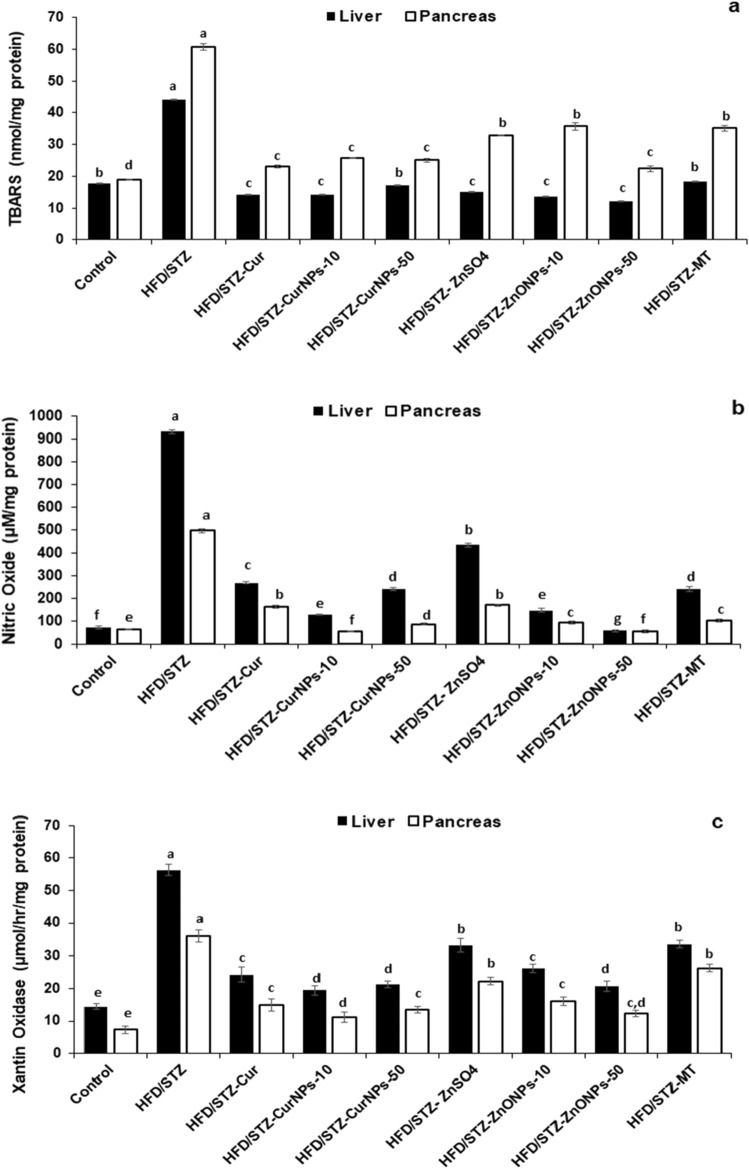

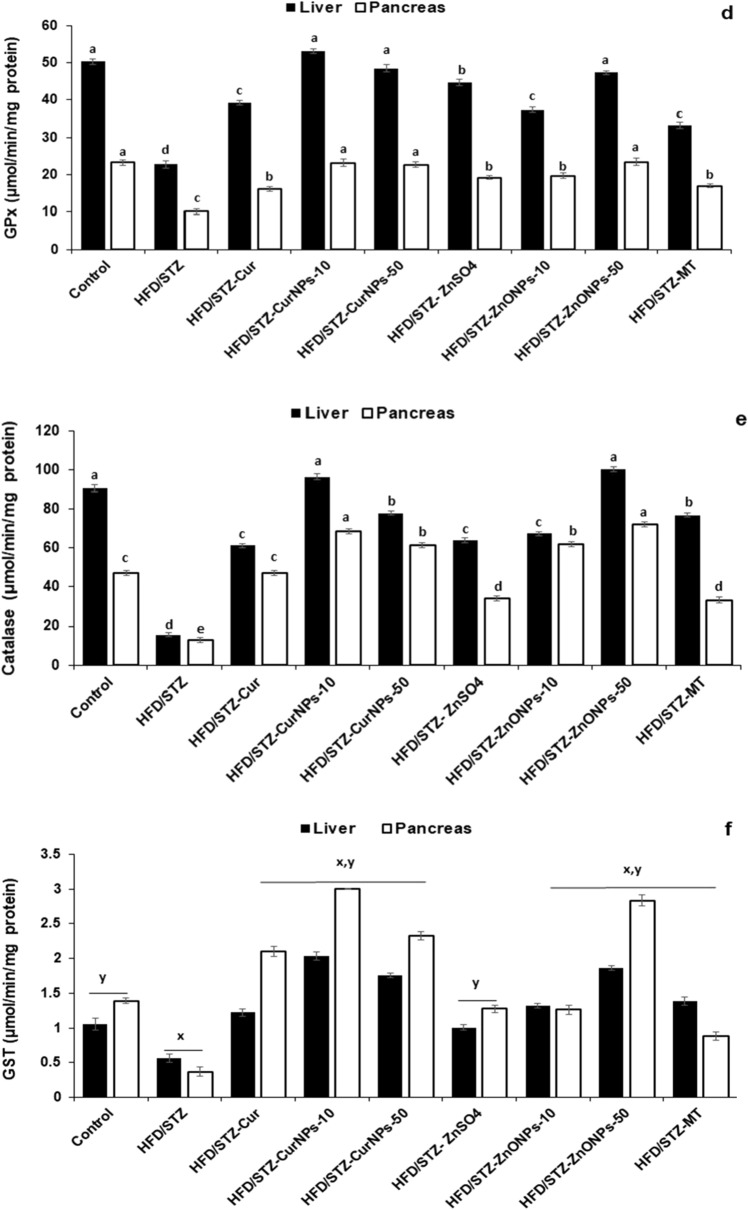

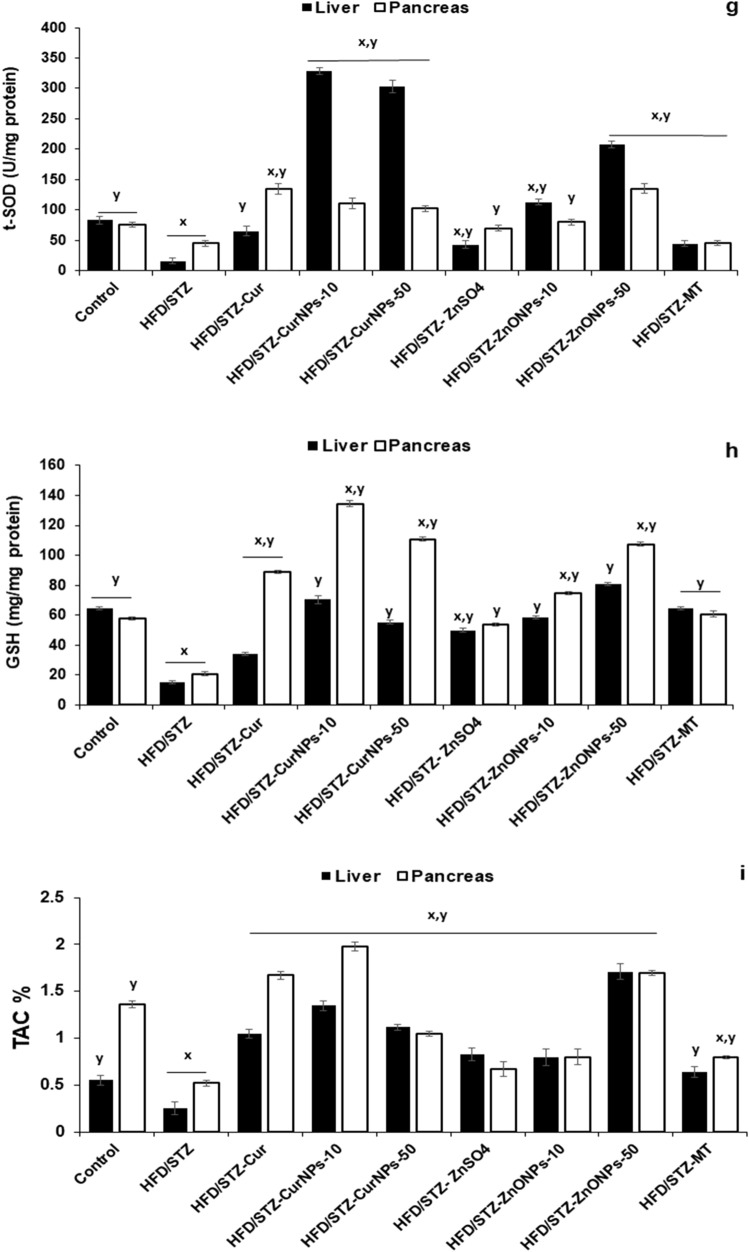
Effect of CurNPs and ZnONPs on change of (**a**) TBARS, (**b**) Nitric oxide and (**c**) Xanthine oxidase levels, (**d**) GPx, (**e**) GST, (**f**) catalase, and (**g**) t-SOD activities, (**h**) GSH and (**i**) TAC levels in liver and pancreas tissues. Values are expressed as mean ± SEM (n = 7), means for the same tissue with different letters in each bar are significantly different (Tukey, *p* < 0.01).

In both liver and pancreas, there were increased TBARS, XO, and NO levels in HFD/STZ-induced rats when related to control healthy animals (*p* < 0.01). Daily administration of CurNPs and ZnONPs as well as conventional curcumin, ZnSO_4,_ and metformin to HFD/STZ-induced rats for 6 weeks produced a significant reduction in TBARS (Fig. 4a), NO (Fig. 4b), and XO (Fig. 4c) levels related to the untreated group (*p* < 0.01). The best results were obtained for groups that received 10 mg/kg CurNPs and 50 mg/kg ZnONPs more than other doses of nanoparticles as well as conventional curcumin and ZnSO_4_. On the other hand, altered activities of antioxidant enzymes suggest increased oxidative stress in HFD/STZ-induced rats. GPx, GST, CAT, and t-SOD (Fig. 4d–g) levels were found to be decreased significantly compared to healthy animals (*p* < 0.01). All current diabetes treatment options produced a significant elevation in hepatic and pancreatic antioxidant enzyme activities compared to the untreated group (*p* < 0.01). Especially, 10 mg/kg CurNPs and 50 mg/kg ZnONPs administration offered better results for antioxidant enzyme activities than other doses of nanoparticles as well as conventional curcumin and ZnSO_4_ to values close to control. Similarly, GSH and TAC levels were found to be decreased in HFD/STZ-induced rats when compared to control animals (*p* < 0.01). Treatment of induction groups with 10 mg/kg CurNPs and 50 mg/kg ZnONPs resulted in a greater increase in GSH (Fig. 4h) and TAC (Fig. 4i) levels than other doses of nanoparticles as well as conventional curcumin and ZnSO_4_ related to the untreated group (*p* < 0.01) and with values higher than control. Either ZnONPs or CurNPs treatment could protect the antioxidant enzymes in hepatic and pancreatic tissues against the harmful effect of HFD and STZ.

### Effect of CurNPs and ZnONPs on serum Adipokines levels of HFD/STZ-induced rats

**Figure 5:**
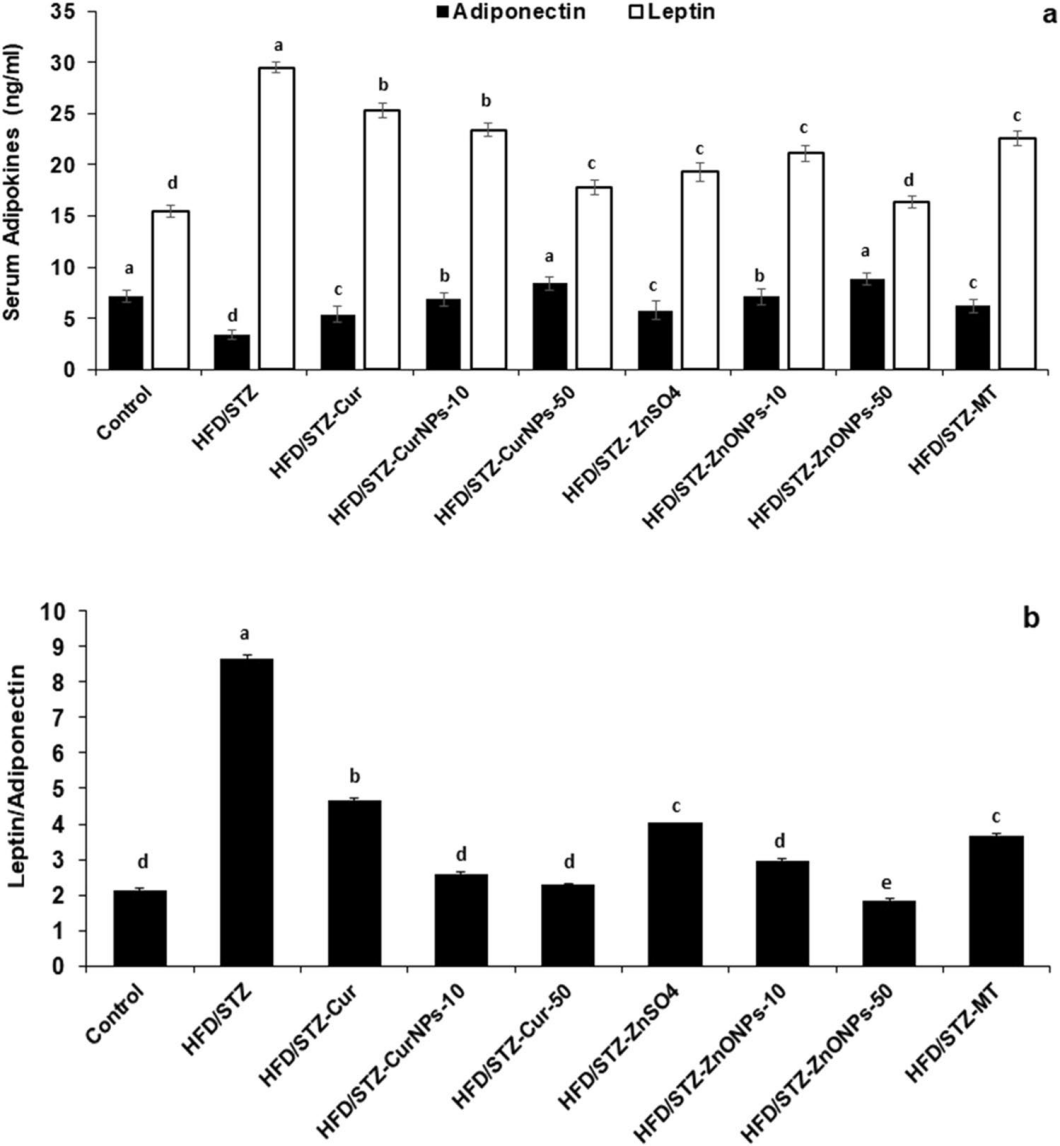
Effect of CurNPs and ZnONPs on change of (**a**) serum Leptin and Adiponectin, (**b**) Leptin/Adiponectin ratio in HFD/STZ-induced rats. Values are expressed as mean ± SEM (n = 7), means for the same tissue with different letters in each bar are significantly different (Tukey, *p* < 0.01).

Serum leptin level was significantly higher in HFD/STZ-induced rats compared to control animals (*p* < 0.01). On the other hand, serum adiponectin level was decreased significantly in HFD/STZ-induced rats as related to control animals (*p* < 0.01). Administration of two doses of CurNPs and ZnONPs as well as conventional curcumin, ZnSO_4,_ and metformin to HFD/STZ-induced rats produced a significant reduction in leptin level and a significant elevation in adiponectin level (Fig. 5a) compared to the untreated group (*p* < 0.01). Groups treated with 10 mg/kg CurNPs and 50 mg/kg ZnONPs; showed a more potent effect on serum adipokines levels than other doses of nanoparticles as well as conventional curcumin and ZnSO_4_ with values near to control. Furthermore, Leptin/Adiponectin ratio was decreased significantly after treatment related to the untreated rats (*p* < 0.01) (Fig. 5b).

### CurNPs and ZnONPs attenuate the inflammation in hepatic and pancreatic tissues of HFD/STZ-induced diabetes

**Figure 6:**
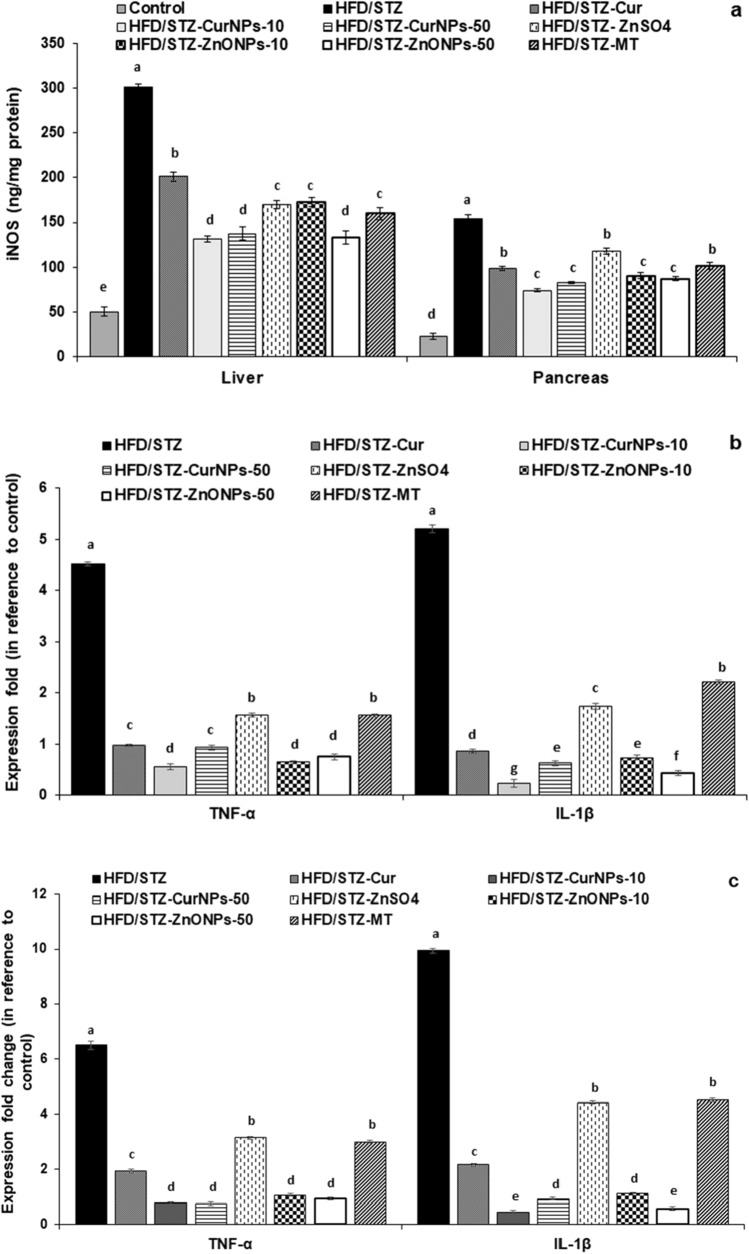
Inhibition of hepatic and pancreatic inflammatory response. (**a**): iNOS levels in liver and pancreas, (**b**): gene expression profile of TNF-α and IL-β in the liver, and (**c**): gene expression profile of TNF-α and IL-β in the pancreas of rats induced by HFD/STZ and treated by CurNPs and ZnONPs. Values are expressed as mean ± SEM (n = 7), means for the same tissue with different letters in each bar are significantly different (Tukey, *p* < 0.01).

To establish the mechanism by which CurNPs and ZnONPs have an anti-inflammatory effect, we next examined the inflammation in the liver and pancreas of induced rats. When compared to control rats, iNOS levels were increased in T2DM-induced rats (*p* < 0.01). Administration of CurNPs and ZnONPs at two doses as well as conventional curcumin, ZnSO_4,_ and metformin was proved to reduce the elevation in iNOS level compared to the untreated group (*p* < 0.01), with a significantly better effect for 10 mg/kg CurNPs and 50 mg/kg ZnONPs administration than other doses of nanoparticles as well as conventional curcumin and ZnSO_4_ (Fig. 6a). Additionally, the inflammatory cytokines gene expression in the liver and pancreas of type 2 diabetic rats induced by HFD/STZ are illustrated. HFD/STZ-induced rats revealed significant up-regulation of gene expression of hepatic and pancreatic inflammatory markers IL-1β and TNF-α when compared to the control group (*p* < 0.01). Interestingly, these elevations in the inflammatory markers were markedly repressed after the administration of CurNPs and ZnONPs as well as conventional curcumin, ZnSO_4,_ and metformin to HFD/STZ-induced group compared to untreated rats (*p* < 0.01). Remarkably, two doses of CurNPs and ZnONPs showed more potent down-regulation of IL-1β and TNF-α expression in relation to other treatments by conventional curcumin and ZnSO_4_ (Fig. 6b and c for liver and pancreas, respectively).

### CurNPs and ZnONPs promote the phosphorylation of the AKT pathway in rats

**Figure 7:**
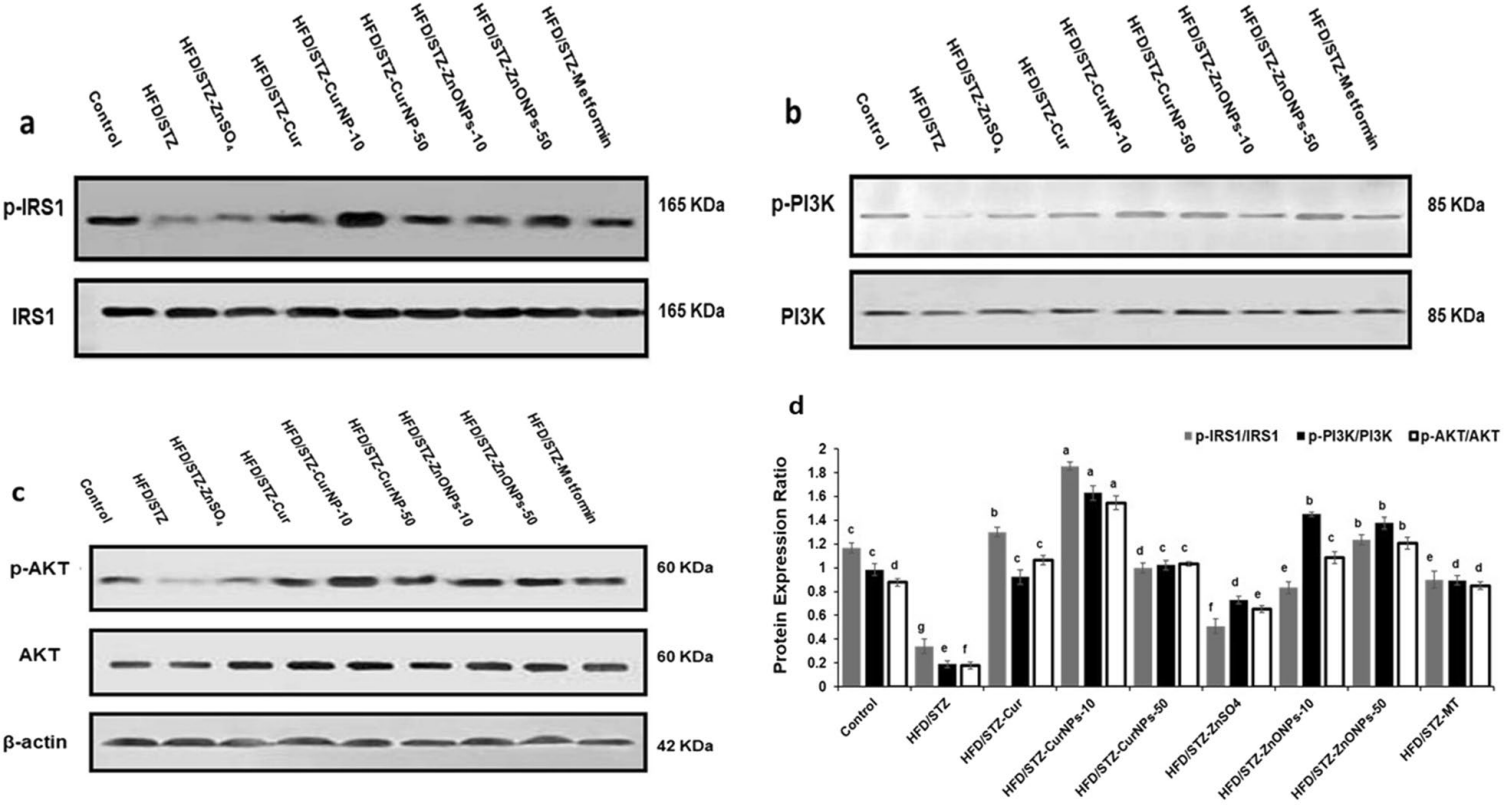
Insulin signaling pathway expression levels by western blot. (**a**) Liver anti-IRS1 & anti-IRS1 pTyr612. (**b**) anti-PI3K & anti-PI3K p85. (**c**) anti-AKT1 & anti-AKT1 pser473 protein levels were measured by western blot. (**d**) Quantitative analysis of p-IRS1/IRS1, p-AKT1/AKT1, and p-PI3K/PI3K in liver tissue of HFD/STZ-induced rats and after treatment with CurNPs and ZnONPs. Values are mean ± SEM (n = 3), means for the same parameter with different letters in each bar are significantly different (Tukey, *p* < 0.01).

According to previous research, the insulin signaling pathway, which includes IRS1, PI3K, and AKT, is a key regulator of glucose uptake through GLUT4. We used western blotting to better understand the underlying molecular mechanism of the influence of CurNPs and ZnONPs in the liver of diabetic rats. As shown in Fig. 7, the HFD/STZ-induced rats had lower phosphorylation levels of IRS1, PI3K, and AKT than the control rats; while this attenuation was ameliorated after administration of both doses of CurNPs and ZnONPs that caused a significant increase (*p* < 0.01) in the phosphorylation of IRS1, PI3K, and AKT in liver tissue compared to untreated rats. CurNPs (10 mg/kg) and ZnONPs (50 mg/kg) showed a more significant rise in phosphorylation levels of insulin signaling members than control values, whereas other treatments showed values that were close to or less than control (Fig. 7a, b).

### CurNPs and ZnONPs reduce the phosphorylation of intermediates in the MAPK pathways in rats

**Figure 8:**
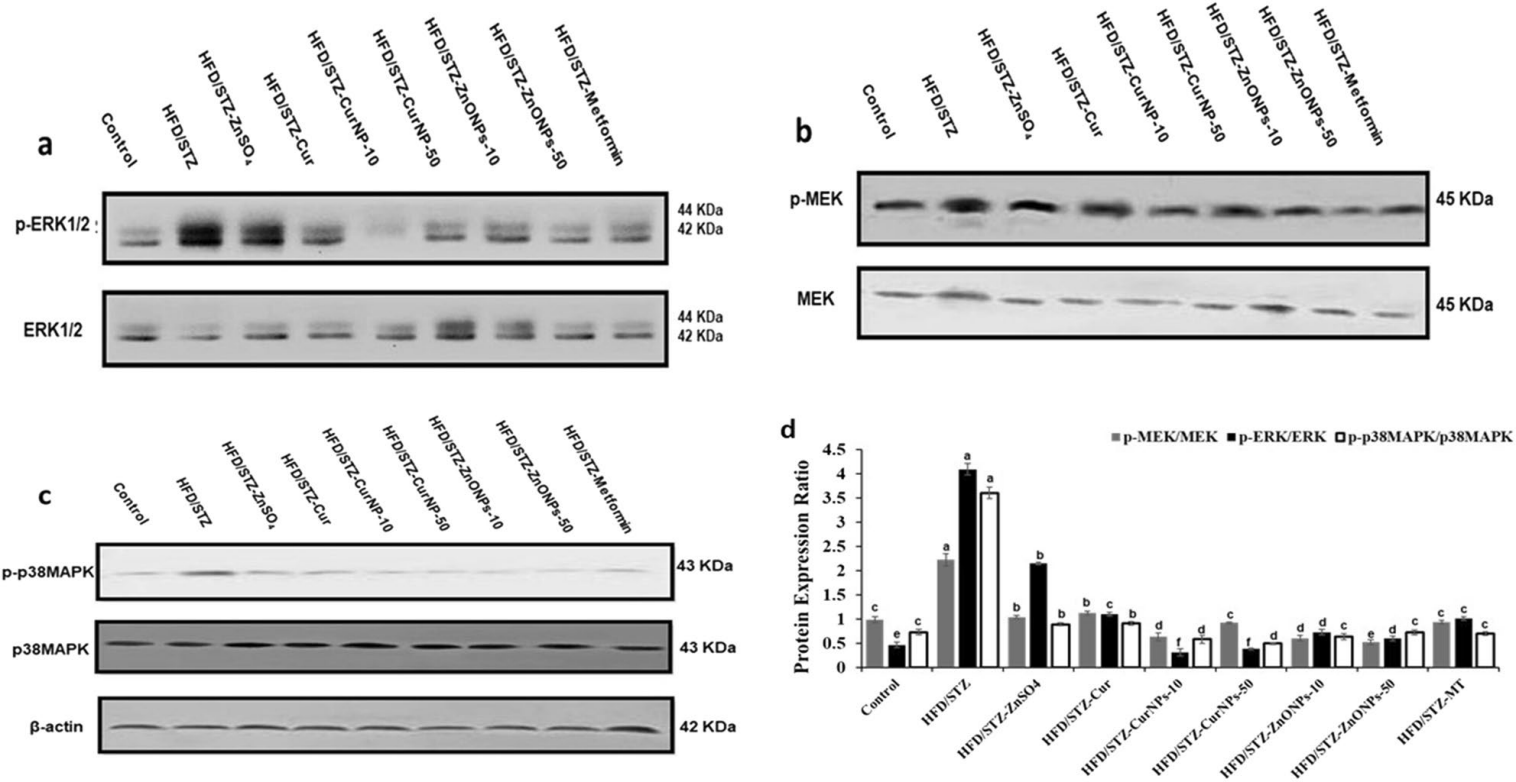
p38-MAPK pathway expression levels by western blot. (**a**) Liver anti ERK1/2 & anti-p-ERK1/2. (**b**) anti-MEK & anti-p-MEK. (**c**) anti-p38MAPK & anti-p-p38MAPK protein levels were measured by western blot. (**d**) Quantitative analysis of p-ERK/ERK, p-MEK/MEK, p-p38MAPK/p38MAPK in liver tissue of HFD/STZ-induced rats and after course treatment with CurNPs and ZnONPs. Values are mean ± SEM (n = 3), means for the same parameter with different letters in each bar are significantly different (Tukey, *p* < 0.01).

MAPK pathways, including the ERK1/2, MEK, and p38-MAPK are involved in the regulation of cell development, oxidative stress, inflammation along with apoptosis, and phosphorylated intermediates in diabetes. In the current study, western blotting was used to determine whether ZnONPs and CurNPs administration decreases the expression of ERK1/2, MEK, and p38-MAPK. The HFD/STZ group showed higher levels of phosphorylation of ERK1/2, MEK, and p38-MAPK in hepatic tissue than the control group. While induced rats administered both doses of ZnONPs and CurNPs showed a significantly better effect in lowering the phosphorylation of ERK1/2, MEK, and p38-MAPK (Fig. 8a–d) in the liver tissue than the other treated groups. It was also observed that ZnONPs and CurNPs lowered the phosphorylation to values less than those in the control groups. These results suggest that both ZnONPs and CurNPs might ameliorate diabetic complications by deactivating MAPK pathways.

### Histopathological analysis of liver

**Figure 9:**
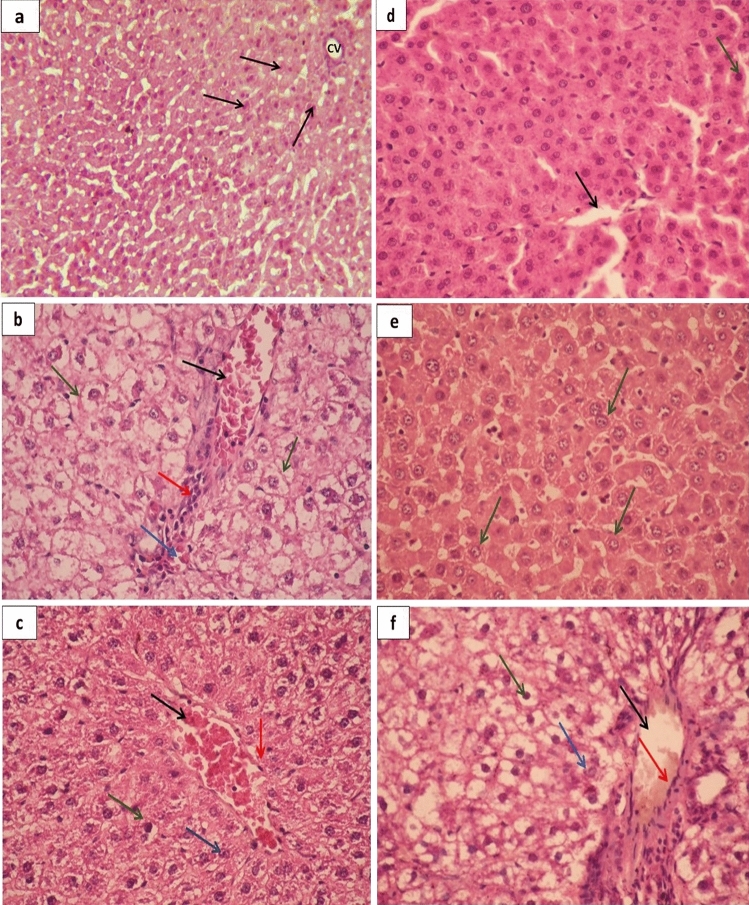

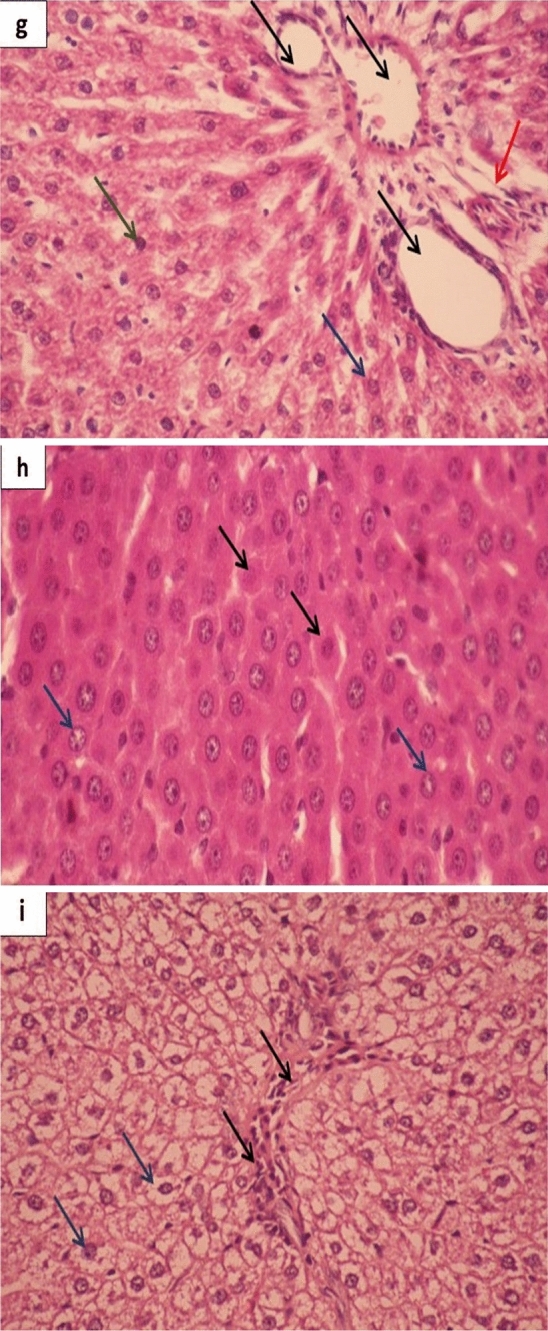
Histopathological study of the liver. (**a**) Photomicrograph of control group displaying liver tissue with normal hepatocytic cords (Black arrows) crowded around a clear central vein (CV) (H&E stains, X400). (**b**) Photomicrograph of HFD/STZ-induced shows a marked dilated hemorrhage portal tract, bile duct (Black arrow), dilated hemorrhage central vein (Blue arrow), and edema. Besides, few infiltrating lymphocytes (Red arrow), and dilation of the sinusoid were seen (vacuolar and hydropic degeneration), which indicate the fatty degeneration of the liver (Green arrows) (H&E stain, X400). (**c**) Photomicrograph of HFD/STZ + Cur shows crowded hepatocyte around the congested portal tract (Black arrow) with few fibrotic cells (Red arrow). Most hepatocytes with rounded nuclei and vacuolated cytoplasm (Green arrows). Many karyolysis hepatocytes and necrotic one was seen (Blue arrow) (H&E stains, X400). (**d**) Photomicrograph of HFD/STZ + CurNPs-10 shows a mild dilated portal tract (Black arrow), the hepatocytes are crowded and have small dark nuclei (Green arrows) (H&E stains, X400). (**e**) Photomicrograph of HFD/STZ + CurNPs-50 shows the necrotic changes of hepatocytes (dark condensed/pyknotic nucleus) (Green arrows) (H&E stains, X400). (**f**) Photomicrograph of HFD/STZ + ZnSO_4_ shows marked dilated portal tract (Black arrow) surrounded with few fibrotic cells (Red arrow), dilated bile duct, and crowded hepatocytes were appeared with round nuclei and vacuolated cytoplasm (Green arrows). With a few necrotic and pyknotic one was seen (Blue arrow) (H&E stains, X400). (**g**) Photomicrograph HFD/STZ + ZnONPs-10 shows marked dilated three portal tracts and bile with the absence of the fibrotic cells (Black arrows). Reorganized hepatocytes architecture with dark rounded nuclei with homogenous non-vacuolated cytoplasm was seen (Green arrows). Few necrotic hepatocytes (Blue arrows) and mildly dilated sinusoid with Kuffer's cell were seen (Red arrows) (H&E stains, X400). (**h**) Photomicrograph of HFD/STZ + ZnONPs-50 shows marked recovering of hepatocytes were appeared with round nuclei containing prominent nucleoli and homogenous non-vacuolated cytoplasm (Black arrows). Few necrotic hepatocyte and pyknotic one was seen (Blue arrows) (H&E stains, X400). (**i**) Photomicrograph of HFD/STZ + MT shows marked dilated and congested portal tract and bile duct surrounded with fibrotic cells (Black arrows). Proliferating hepatocytes were appeared with round nuclei and vacuolated cytoplasm (Blue arrows) (H&E stains, X400).

To study the effect of prepared ZnONPs and CurNPs as well as conventional ZnSO_4_, curcumin, and metformin on histopathological alterations in the liver tissue of induced rats, paraffin sections were stained with hematoxylin and eosin (H&E) for morphological changes in rat liver tissues. Figure 9a displayed the liver of the control group with central vein and normal hepatocytes. On the other hand, the liver of the HFD/STZ-induced group showed a marked dilated hemorrhage portal tract, bile duct, central vein, and edema. Mild dilation of the sinusoid, vacuolar, and hydropic degeneration indicate the fatty degeneration of the liver (Fig. 9b). Moreover, HFD/STZ + Cur treated group revealed a crowded hepatocyte around the congested portal tract with few fibrotic cells, and vacuolated cytoplasm (Fig. 9c). HFD/STZ + CurNPs-10 treated group appeared with mildly dilated two portal tracts and the hepatocytes are crowded and have small dark nuclei (Fig. 9d). As well, HFD/STZ + CurNPs-50 treated group displayed necrotic changes of hepatocytes (dark condensed/pyknotic nucleus) Fig. 9e. The liver of HFD/STZ + ZnSO_4_ treated group showed a marked dilated portal tract surrounded with few fibrotic cells, dilated bile duct, and crowded hepatocytes were appeared with round nuclei and vacuolated cytoplasm (Fig. 9f). Further, HFD/STZ + ZnONPs-10 treated group showed three mild and hemorrhage central veins, reorganized hepatocytes architecture with homogenous cytoplasm (Fig. 9g). While HFD/STZ + ZnONPs-50 treated group exhibited marked recovering of hepatocytes with round nuclei containing prominent nucleoli and homogenous cytoplasm (Fig. 9h). In addition, HFD/STZ + MT treated group showed marked dilated and congested portal tract and bile duct surrounded with fibrotic cells (Fig. 9i).

## Discussion

In the current study, the antidiabetic efficacies of CurNPs and ZnONPs were investigated via the T2DM classical model induced by HFD/STZ. This accepted model was used to investigate the pathophysiology of T2DM. The HFD administration followed by injection of small dose STZ stimulated insulin resistance accompanied by the development of hyperglycemia, hyperinsulinemia, and dyslipidemia in the experimental rats, these results came in agreement with Dolan^36^, who suggested that STZ inhibits glucose metabolism. Our results showed a significant increase in levels of fasting blood glucose and insulin (4 and sevenfold increase, respectively) in addition to an increase in insulin resistance index in untreated diabetic obese rats. While the administration of CurNPs and ZnONPs as well as conventional curcumin, ZnSO_4,_ and metformin significantly enhanced glycemic control via decreasing fasting glucose and insulin levels along with improving insulin sensitivity potentially through the upregulation of the PI3K/AKT pathway, and the downregulation of MAPK pathway activation with greater antidiabetic effect compared to curcumin, ZnSO_4_ as well as metformin. The mechanism by which curcumin provides its hypoglycemic effect has been reported previously. Recent studies by ourselves and others showed that curcumin and zinc can ameliorate hyperglycemia in diabetic rats^20,37^. Also, oral administration of ZnONPs could inhibit the intestinal α-glucosidase enzyme and so reduce glucose absorption and it might enhance glycolysis^19^. It was found that 3 and 10 mg/kg ZnONPs had a much greater antidiabetic effect compared to ZnSO_4_ at dose 30 mg/kg^38^. However, a previous study showed that the short-term administration of 100 mg/kg ZnONPs induced hyperglycemia in healthy and diabetic rats depending on the dose and route of administration^39^. In addition to this, higher blood glucose levels produce tissue damage by several mechanisms; including AGEs formation in the blood, which may be an informative marker for the development of diabetic complications^40^. Our results also showed a significant increase in serum AGEs levels (2.3-fold increase), and these raises were ameliorated significantly by the administration of our current treatment options, with the best effect of 10 mg/kg CurNPs and 50 mg/kg ZnONPs compared to other doses and curcumin, ZnSO_4_ as well as metformin.

The anti-diabetic effect of both CurNPs and ZnONPs was explored by examining the molecular mechanisms underlying insulin resistance developed in HFD/STZ-induced T2DM in rats. Significant repression in levels of p-IRS1/p-PI3K/p-AKT1 was determined in HFD/STZ-induced rats. As the insulin signaling pathway controls the transport of glucose in hepatic cells; dysregulation is a key determinant of the glycemic response showed in uncontrolled diabetes. Previous studies demonstrated that the up-regulation of p-IRS1 and p-AKT might improve the uptake of glucose and reduce blood glucose levels^41^. According to the result of the current study, the protein levels of p-IRS1/p-PI3K/p-AKT increased significantly after oral administration of curcumin and CurNPs, ZnSO_4_ and ZnONPs along with antidiabetic drug metformin in liver cells. However, the effect of CurNPs and ZnONPs on recovering these protein levels was the most powerful among the other therapies.

Also, to establish whether CurNPs and ZnONPs have their effects on glucose levels via MAPK pathways, we measured the phosphorylation of ERK, MEK, and p38-MAPK in the liver of HFD/STZ-induced rats. Earlier work demonstrated that the activation of MAPK pathway might lead to insulin resistance via suppression of IRS phosphorylation^42^. In addition, the MAPK pathway activation leads to suppression of GLUT4 expression, which reduced the transport of glucose^43^. In the present study, the activation of ERK, MEK, and p38 MAPK in the liver of T2D-induced rats was significantly suppressed by the administration of both nanoparticles so that 10 mg/kg CurNPs and 50 mg/kg ZnONPs treatments were more effective compared to other doses and curcumin, ZnSO_4_ as well as metformin. These findings suggest that CurNPs and ZnONPs may increase glucose uptake, ameliorate insulin resistance, and inflammation by reducing phosphorylation of MAPK pathways.

As confirmed before that diabetes is linked with abnormalities in lipid metabolism that are generally characterized by an elevation in the serum lipid levels^44,45^. It has been reported that curcumin and CurNPs can revert the aggravated serum lipid profile through lowering these secondary complications; obesity, adipogenesis, HMG-CoA reductase, cholesterol absorption, and intestinal transmission, lipogenic genes expression, raising LDL receptors and controlling some genes implicated in lipoprotein and lipid metabolism^46,47^. Another study revealed that ZnONPs exhibited a remarkable recovery in TG and TC levels. These anti-hyperlipidemic properties of ZnONPs might be linked with the stimulating insulin-like nature of nanoparticles on the affected pancreatic β cells. Current results confirmed the specific role of CurNPs and ZnONPs along with conventional curcumin and ZnSO_4_ as a lipid profile regulator and come in agreement with our previous work on zinc and curcumin effect on diabetes^20^.

Liver aminotransferases are very sensitive indicators for hepatic injury and toxicity^48^. Our results on liver function enzymes revealed the beneficial effects of current diabetes treatments on serum aminotransferases. Remarkably, the kidney has been the center of much of the study involving the common complications associated with diabetes. In our study, elevated levels of serum urea, and creatinine were showed in HFD/STZ-induced rats, which are perfect indicators of renal dysfunction^49^. The detected renal dysfunction in HFD/STZ- induced rats that occurring at the end of 8 weeks was reversed by treatment with nanoparticles and conventional treatments.

The progression of DM was associated with an imbalance in the cellular process between oxidative and anti-oxidative systems, which arises through the excess production of ROS. SOD, CAT, GPx, and GST are the most important antioxidant enzymes, which can remove ROS and prohibit the toxic effects of oxidant molecules on tissues and cells^50^. In both hepatic and pancreatic tissues, our results of diabetic rats established that our current diabetes treatment options; particularly 10 mg/kg CurNPs and 50 mg/kg ZnONPs, effectively restored pancreatic and hepatic antioxidant enzyme activities along with decreasing oxidative stress markers; MDA and NO. Importantly, curcumin can restore pancreatic islets by enhancing islet viability and inhibited islet ROS production, and boost body antioxidant activity^51,52^. While the ameliorative effect of curcumin on hepatic GSH levels was attached to the ability of curcumin to scavenge free radicals through interacting with the oxidative cascade to reduce oxidative enzymes, return the antioxidant status, and chelate metal ions; thus avoiding the Fenton reaction^53^. Consistent with our results, a former study described that dietary zinc supplementation to diabetic rats could increase catalase and GPx activities as well as GSH levels in the liver of diabetic rats, so it has a beneficial effect on oxidative stress^54,55^. The obtained data revealed that the treatment with 10 mg/kg CurNPs and 50 mg/kg ZnONPs indicated a more beneficial effect against the development of T2DM by increasing hepatic and pancreatic antioxidant enzyme activities and non-enzymatic antioxidants associated with a significant reduction in oxidative stress markers in T2DM-induced rats compared to other doses of nanoparticles, curcumin, ZnSO_4_ as well as metformin. In contrast to our current work, a previous study observed an elevated level of MDA particularly at higher doses of ZnONPs accompanied by altered antioxidant enzyme activities. Indeed, the amount of ROS generation by engineered nanomaterials is associated with the chemical nature of the nanoparticles; size and high specific surface area might lead to the production of ROS and induction of oxidative damages^38^.

Usually, obesity disturbs the inflammation balance, which increases the level of proinflammatory cytokines^56^. Inflammation can cause peripheral insulin resistance and hyperglycemia^57^. Notably, among current diabetes treatment options used in our study; 10 mg/kg CurNPs and 50 mg/kg ZnONPs exhibited more anti-inflammatory power and showed significant suppression of expression levels of TNF-α, IL-1β as well as iNOS concentration in liver and pancreas. This may be due to the cytokines regulatory effect of curcumin through decreasing expression and liberation of inflammatory cytokines^58^. Too, CurNPs safely prevent STZ-induced oxidative stress and inflammation in pancreatic beta cells^59^. Also, these data come in agreement with that in a previous study showing the similar anti-inflammatory effect of ZnONPs on downregulating the expression of iNOS, IL-1β, and TNF-α in the cell^60^.

Likewise, adiponectin acts as an anti-inflammatory cytokine, which plays an important role in the pathogenesis of T2DM^61^. Curcumin could reduce inflammation by downregulating other inflammatory cytokines, such as TNF-α, leptin, and increases adiponectin levels^62^. Additionally, Adachi et al. presented that zinc supplementation could reduce the insulin level, glucose tolerance, insulin resistance, and increased depressed adiponectin levels^63^. Our data revealed a significant decrease in adiponectin (2.3-fold) and an increase in leptin levels (1.9-fold) in HFD/STZ induced rats. While supplementation of curcumin, ZnSO_4_, two doses of CurNPs and ZnONPs as well as metformin in our study, can specifically improve these disturbances in Adipokines levels.

The histopathological results were coming with the biochemical and molecular examinations. The liver tissue micro-architecture was strictly damaged in HFD/STZ-induced rats. The ameliorating effects of CurNPs and ZnONPs on liver tissue showed in the histopathological study indicate restoration of the architecture of the cells. These mild, moderate, and marked recoveries may be due to the ability of these nanoparticles to decrease inflammation and oxidative stress. The present finding revealed a better outcome in rats treated with 10 mg/kg CurNPs and 50 mg/kg ZnONPs that exhibited a mild restoration of the hepatocytes structure along with amelioration of liver function biomarkers indicating their hepatoprotective and therapeutic effects. These effects could be mediated by the potent antioxidant and anti-inflammatory activities of prepared nanoparticles^64,65^. These results pointed out the possibility of prepared nanoparticles to reduce or treat complications of diabetes.

## Conclusion

**Figure 10:**
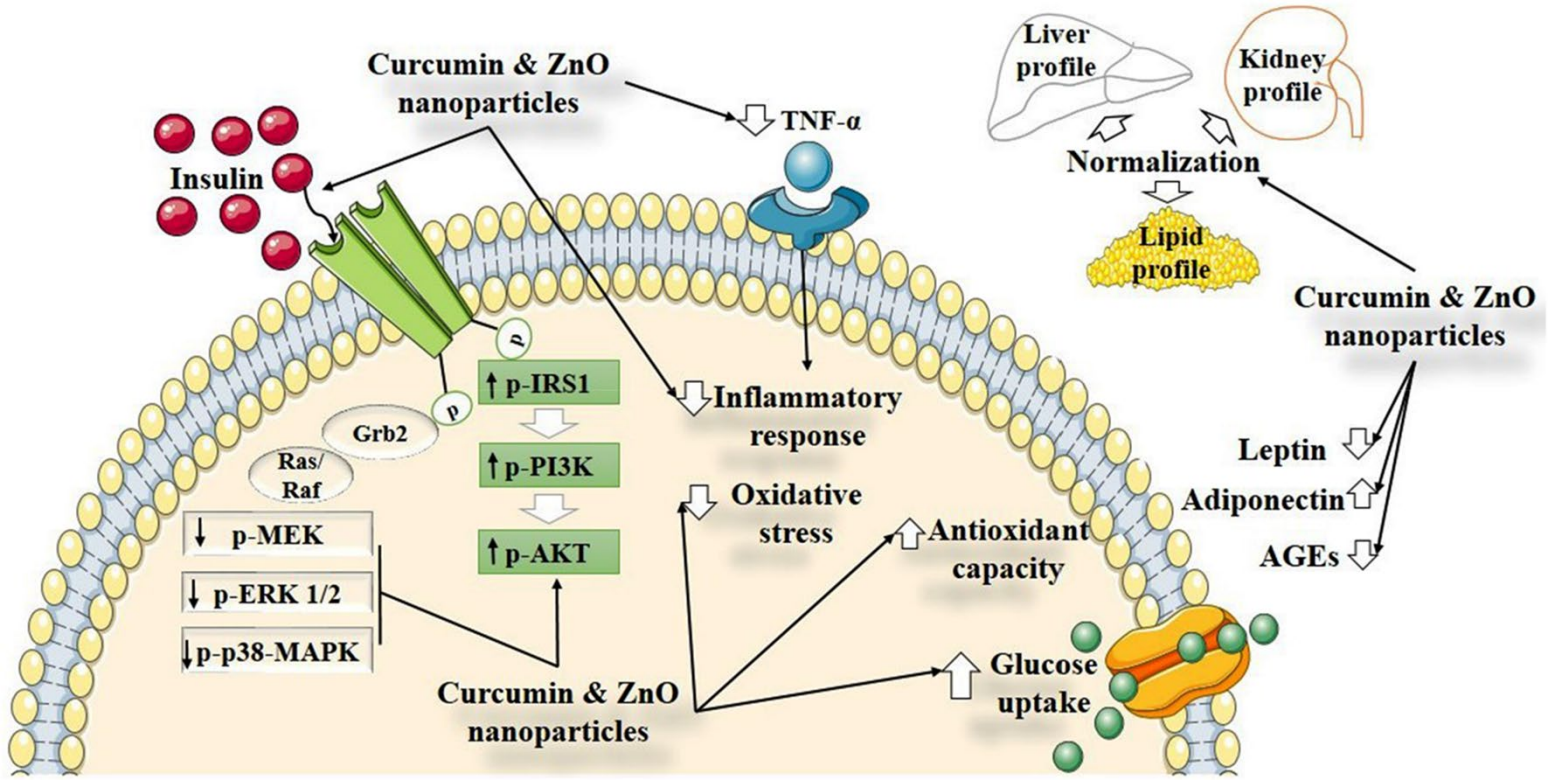
Schematic illustration for the effects of ZnONPs and CurNPs on pathways-related T2DM. HFD/STZ induced hyperglycemia, dyslipidemia, cellular stress, insulin resistance, causing the inhibition of PI3K/AKT and activation of p38-MAPK. ZnONPs and CurNPs administration displayed protective mechanisms at cellular and molecular levels. Both nanoparticles could protect tissues from diabetic complications via upregulation of PI3K/AKT pathway, down-regulation of p38-MAPK pathways, and attenuation of oxidative and inflammatory pathways. (↑): Up-regulated targets; (↓) Down-regulated targets.

This study indicates that 10 mg/kg CurNPs and 50 mg/kg ZnONPs have a more efficient anti-diabetic effect than conventional curcumin and ZnSO_4_ as well as an anti-diabetic drug, metformin on all measured parameters. As shown by the reduction in the serum levels of glucose and insulin, likely mediated through the upregulation of PI3K/AKT signaling pathway in the hepatic tissue. More interestingly, they ameliorate the clinical signs of diabetes as inflammation, oxidative stress, and insulin resistance via inhibition of MAPK pathways along with a noticeable alleviation of the structural damage of the liver. This might be the first report to point out the effect of CurNPs and ZnONPs on pathways related to insulin signaling in T2DM. From the results of the current study and our previous study, we can conclude that the incorporation of nanoparticles in the therapeutic strategies would provide new avenues to increase their potency against chronic disorders like T2DM. A Schematic illustration of the effects of ZnONPs and CurNPs on the different pathways is shown in Fig. 10.

## Supplementary Information

Supplementary Information.
