# Supplementary material for: Ameliorative effect of curcumin and zinc oxide nanoparticles on multiple mechanisms in obese rats with induced type 2 diabetes
Source: Sci Rep. 2021 Oct 19;11:20677. doi: 10.1038/s41598-021-00108-w (PMC8526574; doi:10.1038/s41598-021-00108-w)
Supplement: Supplementary file 1 — Supplementary Information. [file 41598_2021_108_MOESM1_ESM.docx]

**Ameliorative effect of curcumin and zinc oxide nanoparticles on multiple mechanisms in obese rats with induced type 2 diabetes**

**Short running title:**

**Type 2 diabetes and potential use of curcumin and Zinc oxide nanoparticles**

Shaymaa Abdulmalek^1,2^, Asmaa Eldala^1^, Doaa Awad^1^, Mahmoud Balbaa^1,#^

^1^Department of Biochemistry, Faculty of Science, Alexandria University, Alexandria 21511, Egypt, ^2^ Center of Excellency for Preclinical Study (CE-PCS), Pharmaceutical and Fermentation Industries Development Centre, the City of Scientific Research and Technological Applications, SRTA-City, New Borg El-Arab City, Alexandria, Egypt.

#Corresponding author: Fax: ‏+20 3 3911794; ORCID number: http://orcid.org/0000-0002-0876-6604).

**Email address:**

shimaa_salamy@yahoo.com (S. A. Abdulmalek)

[asmaafathy451@gmail.com](mailto:asmaafathy451@gmail.com) (A. Eldala)

doaaelsayed363@hotmail.com (D. El Sayed)

mahmoud.balbaa@alexu.edu.eg (M. Balbaa)

**HFD/STZ-Metformin**

**HFD/STZ-ZnONPs-50**

**HFD/STZ-ZnONPs-10**

**HFD/STZ-CurNP-50**

**HFD/STZ-CurNP-10**

**HFD/STZ-Cur**

**HFD/STZ-ZnSO_4_**

**HFD/STZ**

**Control**

**70 KDa**


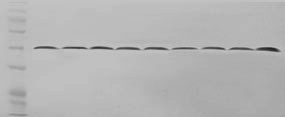


**β-actin**

**24 KDa**

**31 KDa**

**42 KDa**

**55 KDa**

**HFD/STZ-Metformin**

**HFD/STZ-ZnONPs-50**

**HFD/STZ-ZnONPs-10**

**HFD/STZ-CurNP-50**

**HFD/STZ-CurNP-10**

**HFD/STZ-Cur**

**HFD/STZ-ZnSO_4_**

**HFD/STZ-Metformin**

**HFD/STZ-ZnONPs-50**

**HFD/STZ-ZnONPs-10**

**HFD/STZ-CurNP-50**

**HFD/STZ-CurNP-10**


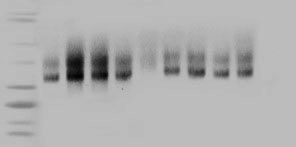

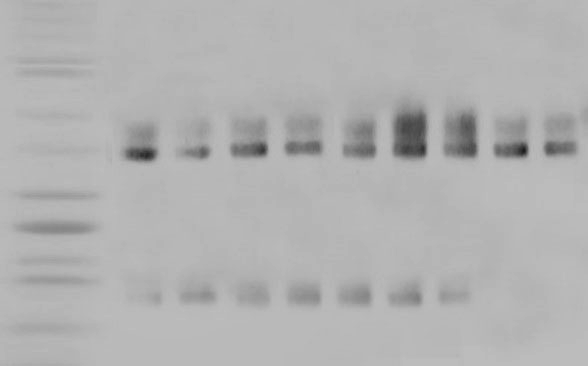


**p-ERK1/2 (1)**

**HFD/STZ**

**Control**

**HFD/STZ-Cur**

**HFD/STZ**

**HFD/STZ-ZnSO_4_**

**Control**

**ERK1/2**

**70 KDa**

**31 KDa**

**42 KDa**

**44 KDa**

**HFD/STZ-CurNP-10**

**HFD/STZ-CurNP-50**

**HFD/STZ-ZnSO_4_**

**HFD/STZ-ZnONPs-10**

**HFD/STZ-ZnONPs-50**

**HFD/STZ-Metformin**


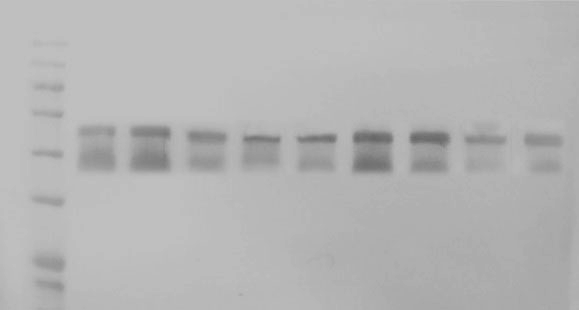


**Control**

**HFD/STZ**

**HFD/STZ-Cur**

**p-ERK1/2 (2)**

**HFD/STZ-ZnONPs-50**

**HFD/STZ-ZnONPs-10**

**HFD/STZ-CurNP-50**

**HFD/STZ-CurNP-10**

**HFD/STZ-Metformin**

**HFD/STZ-ZnONPs-50**

**HFD/STZ-CurNP-10**

**HFD/STZ-CurNP-50**

**HFD/STZ-ZnONPs-10**


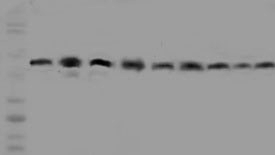

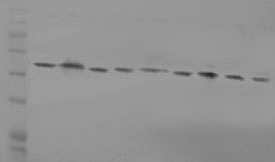


**MEK**

**p-MEK (1)**

**HFD/STZ-Metformin**

**HFD/STZ-Cur**

**HFD/STZ-ZnSO_4_**

**HFD/STZ**

**Control**

**Control**

**HFD/STZ**

**HFD/STZ-ZnSO_4_**

**HFD/STZ-Cur**

**42 KDa**

**45 KDa**

**70 KDa**


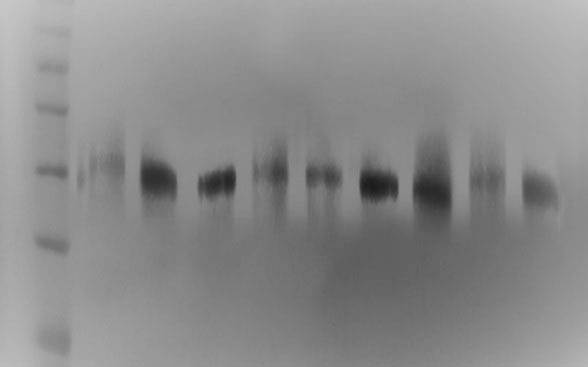


**p-MEK (2)**

**HFD/STZ-Metformin**

**HFD/STZ-ZnONPs-50**

**HFD/STZ-ZnONPs-10**

**HFD/STZ-CurNP-50**

**HFD/STZ-CurNP-10**

**HFD/STZ-Cur**

**HFD/STZ-ZnSO_4_**

**HFD/STZ**

**Control**


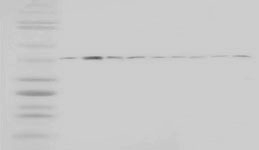

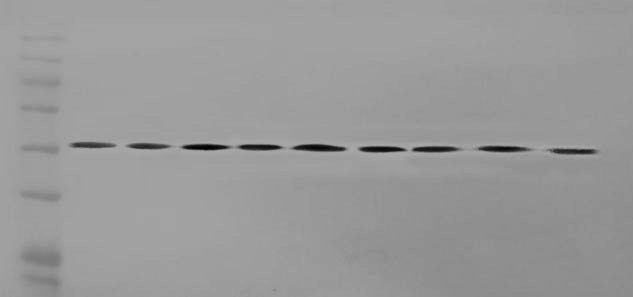


**HFD/STZ-ZnONPs-10**

**MAPK**

**p-MAPK**

**HFD/STZ-Metformin**

**HFD/STZ-ZnONPs-50**

**HFD/STZ-CurNP-50**

**HFD/STZ-CurNP-10**

**HFD/STZ-Cur**

**HFD/STZ-ZnSO_4_**

**HFD/STZ**

**Control**

**HFD/STZ-Metformin**

**HFD/STZ-ZnONPs-50**

**HFD/STZ-ZnONPs-10**

**HFD/STZ-CurNP-50**

**HFD/STZ-CurNP-10**

**HFD/STZ-Cur**

**HFD/STZ-ZnSO_4_**

**HFD/STZ**

**Control**

**70 KDa**

**45 KDa**

**42 KDa**


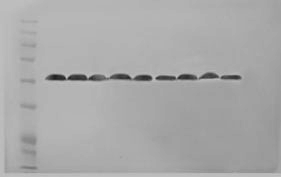

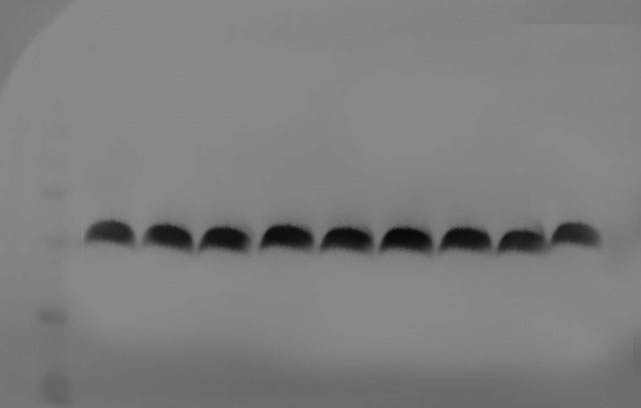


**β-actin (2)**

**β-actin (1)**

**HFD/STZ-Metformin**

**HFD/STZ-Cur**

**HFD/STZ-CurNP-10**

**HFD/STZ-CurNP-50**

**HFD/STZ-ZnONPs-10**

**HFD/STZ-ZnONPs-50**

**HFD/STZ**

**HFD/STZ-ZnSO_4_**

**Control**

**42 KDa**

**HFD/STZ-Metformin**

**HFD/STZ-ZnONPs-50**

**HFD/STZ-ZnONPs-10**

**HFD/STZ-CurNP-50**

**HFD/STZ-Cur**

**HFD/STZ-CurNP-10**

**HFD/STZ-ZnSO_4_**

**Control**

**HFD/STZ**

**70 KDa**

**HFD/STZ-Metformin**

**HFD/STZ-CurNP-10**

**HFD/STZ-CurNP-50**

**HFD/STZ-ZnONPs-10**

**HFD/STZ-ZnONPs-50**

**HFD/STZ-ZnSO_4_**

**HFD/STZ-ZnONPs-10**

**HFD/STZ-CurNP-50**

**HFD/STZ-Cur**

**HFD/STZ**

**Control**

**HFD/STZ-Metformin**

**HFD/STZ-ZnONPs-50**

**HFD/STZ-CurNP-10**

**HFD/STZ-Cur**

**HFD/STZ-ZnSO_4_**


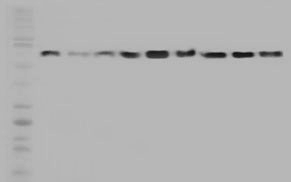

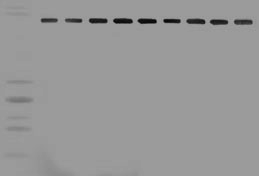


**p-AKT**

**AKT**

**HFD/STZ**

**Control**

**60 KDa**

**70 KDa**


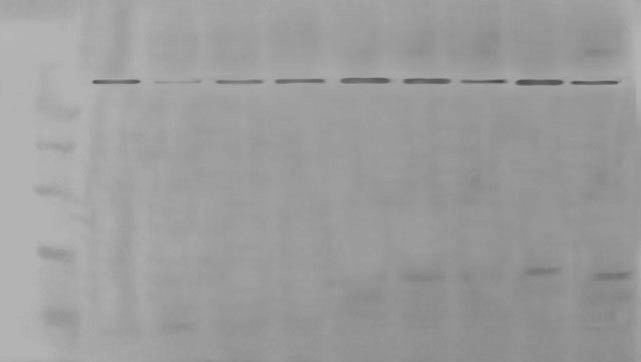

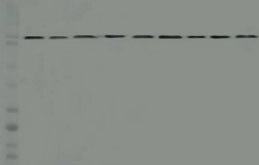


**HFD/STZ-ZnONPs-50**

**HFD/STZ-Metformin**

**PI3K**

**p-PI3K**

**Control**

**HFD/STZ**

**HFD/STZ-ZnSO_4_**

**HFD/STZ-Cur**

**HFD/STZ-CurNP-10**

**HFD/STZ-CurNP-50**

**HFD/STZ-ZnONPs-10**

**HFD/STZ-Metformin**

**HFD/STZ-ZnONPs-50**

**HFD/STZ-ZnONPs-10**

**HFD/STZ-CurNP-50**

**HFD/STZ-CurNP-10**

**HFD/STZ-Cur**

**HFD/STZ-ZnSO_4_**

**HFD/STZ**

**Control**

**85 KDa**

**70 KDa**


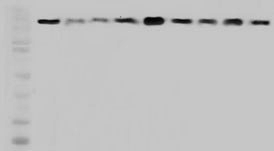

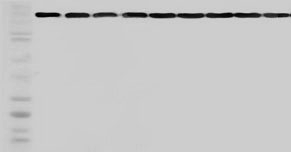


**165 KDa**

**IRS**

**p-IRS**

**HFD/STZ-Metformin**

**HFD/STZ-Metformin**

**HFD/STZ-ZnONPs-50**

**HFD/STZ-ZnONPs-50**

**HFD/STZ-ZnONPs-10**

**HFD/STZ-ZnONPs-10**

**HFD/STZ-CurNP-50**

**HFD/STZ-CurNP-50**

**HFD/STZ-CurNP-10**

**HFD/STZ-CurNP-10**

**HFD/STZ-Cur**

**HFD/STZ-Cur**

**HFD/STZ-ZnSO_4_**

**HFD/STZ-ZnSO_4_**

**HFD/STZ**

**HFD/STZ**

**Control**

**Control**

**70 KDa**

**Supplementary Figure 1. Western blots for β-actin, p-ERK1/2, ERK1/2, p-MEK, MEK, p-MAPK, MAPK, β-actin, p-AKT, AKT, p-PI3K, PI3K, p-IRS, and IRS.**
